# Three-dimensional photonic quantum Hall effect of Fermi arcs

**DOI:** 10.1093/nsr/nwag251

**Published:** 2026-04-29

**Authors:** Zhengting Wu, Minqi Cheng, Ziyao Wang, Siqi Xu, Jingming Chen, Yan Meng, Xiang Xi, Xiankai Sun, Perry Ping Shum, Haizhou Lu, Zhen Gao

**Affiliations:** State Key Laboratory of Optical Fiber and Cable Manufacturing Technology, Department of Electronic and Electrical Engineering, Guangdong Key Laboratory of Integrated Optoelectronics Intellisense, Southern University of Science and Technology, Shenzhen 518055, China; State Key Laboratory of Optical Fiber and Cable Manufacturing Technology, Department of Electronic and Electrical Engineering, Guangdong Key Laboratory of Integrated Optoelectronics Intellisense, Southern University of Science and Technology, Shenzhen 518055, China; State Key Laboratory of Optical Fiber and Cable Manufacturing Technology, Department of Electronic and Electrical Engineering, Guangdong Key Laboratory of Integrated Optoelectronics Intellisense, Southern University of Science and Technology, Shenzhen 518055, China; State Key Laboratory of Optical Fiber and Cable Manufacturing Technology, Department of Electronic and Electrical Engineering, Guangdong Key Laboratory of Integrated Optoelectronics Intellisense, Southern University of Science and Technology, Shenzhen 518055, China; State Key Laboratory of Optical Fiber and Cable Manufacturing Technology, Department of Electronic and Electrical Engineering, Guangdong Key Laboratory of Integrated Optoelectronics Intellisense, Southern University of Science and Technology, Shenzhen 518055, China; School of Electrical Engineering and Intelligentization, Dongguan University of Technology, Dongguan 523808, China; School of Electrical Engineering and Intelligentization, Dongguan University of Technology, Dongguan 523808, China; Department of Electronic Engineering, The Chinese University of Hong Kong, Hong Kong 999077, China; State Key Laboratory of Optical Fiber and Cable Manufacturing Technology, Department of Electronic and Electrical Engineering, Guangdong Key Laboratory of Integrated Optoelectronics Intellisense, Southern University of Science and Technology, Shenzhen 518055, China; State Key Laboratory of Quantum Functional Materials, Department of Physics, and Guangdong Basic Research Center of Excellence for Quantum Science, Southern University of Science and Technology, Shenzhen 518055, China; Quantum Science Center of Guangdong-Hong Kong-Macao Greater Bay Area (Guangdong), Shenzhen 518045, China; State Key Laboratory of Optical Fiber and Cable Manufacturing Technology, Department of Electronic and Electrical Engineering, Guangdong Key Laboratory of Integrated Optoelectronics Intellisense, Southern University of Science and Technology, Shenzhen 518055, China

**Keywords:** quantum Hall effect of Fermi arcs, gyromagnetic photonic crystals, chiral hinge states

## Abstract

The recent discovery of three-dimensional (3D) quantum Hall effect (QHE) of Fermi arcs in topological semimetals has revolutionized our understanding of Hall physics in 3D systems. However, its most prominent hallmark, the one-sided chiral hinge states of Fermi arcs, has thus far never been experimentally observed in any physical system. Here, we report the first photonic realization of 3D QHE of Fermi arcs and directly observe the one-sided chiral hinge states of Fermi arcs in an inhomogeneous magnetic Weyl photonic crystal under a pseudomagnetic field (PMF) with time-reversal symmetry breaking. We experimentally demonstrate that the PMF quantizes both the bulk and Fermi arc surface states into Landau plateaus, giving rise to chiral Landau levels and robust one-sided chiral hinge states localized at only one edge on the front surface and at the opposite edge on the back surface, both of which are the signatures of 3D QHE of Fermi arcs. Moreover, we show that the one-sided chiral hinge states of Fermi arcs can be switched between the two pairs of diagonal hinges by reversing the PMF. Our work not only provides an ideal platform for exploring 3D quantum Hall physics but also opens new avenues for the design of robust photonic devices.

## Teaser

This work reports the first experimental observation of the three-dimensional photonic quantum Hall effect of Fermi arcs and its one-sided chiral hinge states in an inhomogeneous magnetic Weyl photonic crystal.

## INTRODUCTION

**Figure 1.**
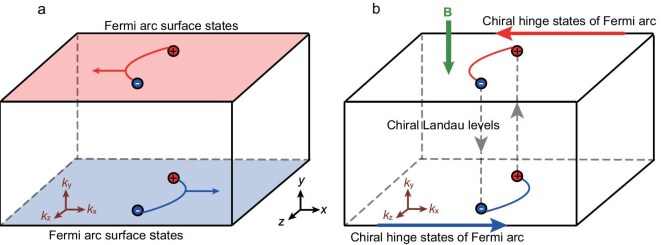
Three-dimensional QHE of Fermi arcs in Weyl semimetals. (a) Fermi arc surface state of Weyl semimetals. Oppositely charged WPs (red and blue dots) are connected by surface Fermi arcs (red and blue arcs) on the opposite surfaces. The arrows denote the propagation directions of the chiral Fermi arc surface states. (b) Under a magnetic field (or PMF) B (green arrow), the bulk states and the closed Fermi loops composing two surface Fermi arcs give rise to chiral Landau levels (gray dashed lines with arrows) and one-sided chiral hinge states of Fermi arcs (red and blue arrows) at two diagonal hinges, which are the hallmark signatures of 3D QHE of Fermi arcs.

Weyl semimetals [1,2], three-dimensional (3D) topological states of matter with charged Weyl points (WPs), have attracted great attention from condensed matter [3–7] to classical wave [8–18] physics. One hallmark of Weyl semimetals is the surface Fermi arcs, which connect pairs of projected WPs with opposite topological charge (red and blue dots) and form open arcs (red and blue arcs) in the surface Brillouin zone (BZ), as illustrated in Fig. 1a. Under a natural [19–23] or artificial [24–29] magnetic field, Weyl semimetals host chiral Landau levels (chiral zero modes) [27–29] with linear dispersion determined by the chirality of the WPs and the direction of the magnetic field, which underlies many intriguing phenomena such as chiral anomaly [24–26], negative longitudinal magnetoresistance [30,31], and planar Hall effect [32–34].

On the other hand, since the discovery of two-dimensional (2D) quantum Hall effect (QHE) [35,36], tremendous efforts have been made to extend it to three dimensions [37–41], which is, however, extremely challenging because the extra dimension along the magnetic field prevents the opening of the bulk band gap and the quantization of Hall conductance [42]. Recently, a novel 3D QHE of Fermi arcs [43] was discovered in Weyl semimetals under a magnetic field (green arrow) with time-reversal symmetry (TRS) breaking, in which the open Fermi arcs (red and blue arcs) at two opposite surfaces can form a closed Fermi loop via a “wormhole” tunneling through the Weyl nodes (red and blue dots) and chiral Landau levels (gray dashed arrows) and support a 3D QHE [44], as illustrated in Fig. 1b. More intriguingly, this exotic 3D QHE supports one-sided chiral hinge states of Fermi arcs (red and blue arrows), exhibiting a higher-order topological phase of matter that contrasts with all previous QHE systems [35,41,45–50] and their classical analogues [51–53], which usually support first-order chiral edge (surface) states in 2D (3D) systems. To date, the 3D QHE of Fermi arcs has been experimentally demonstrated in Dirac semimetals Cd_3_As_2_ [54–61], which, however, is questionable and may arise from trivial mechanisms such as a complete 2D electron gas on a single surface or 3D bulk states quantizing to 2D sub-bands [62]. Most notably, Dirac semimetals consist of two time-reversed Weyl semimetals, resulting in two-sided chiral hinge states on both sides of the top and bottom surfaces [63], rather than the desired one-sided chiral hinge states on one side of the top surface and the opposite side of the bottom surface. More recently, the 3D QHE of Fermi arcs has also been experimentally explored in 3D acoustic Weyl crystals [64] and synthetic dimensions [65,66]. However, due to the TRS of these acoustic and elastic wave systems, the one-sided topological hinge states observed are nonchiral, rather than chiral (see the tight-binding (TB) results of the system with TRS in Note S1). Therefore, so far, the one-sided chiral hinge states of Fermi arcs, which are the hallmark feature of 3D QHE of Fermi arcs, have never been directly observed in any physical system (including condensed matter and classical wave systems), not to mention manipulating them.

Here, we report the first 3D photonic QHE of Fermi arcs in an inhomogeneous magnetic Weyl photonic crystal with TRS breaking, and directly observe one-sided chiral hinge states of Fermi arcs located at one edge of the front surface and the opposite edge of the back surface. Unlike in electronic systems, where a single magnetic field can simultaneously break TRS and induce Landau quantization, a magnetic field has no direct effect on electromagnetic waves. Therefore, we introduce a structural gradient in a magnetic Weyl photonic crystal—in which a real magnetic field is used to break TRS and create the magnetic Weyl semimetal phase—to shift the WPs. This constructs an in-plane pseudomagnetic field (PMF) that affects the electromagnetic waves, analogous to the effect of a real magnetic field on electrons. The PMF, perpendicular to the surface Fermi arcs on opposite surfaces, quantizes both the bulk and Fermi arc surface states into Landau level plateaus and gives rise to chiral zeroth bulk modes and one-sided chiral hinge states of Fermi arcs, both of which are hallmark features of the 3D QHE of Fermi arcs. Moreover, we show that the one-sided chiral hinge states of the Fermi arcs can be switched between the two pairs of diagonal hinges by reversing the PMF or changing the energy. We experimentally demonstrate that the one-sided chiral hinge states of Fermi arcs are robust against metallic obstacles, with potential applications in 3D integrated photonic circuits.

### Three-dimensional inhomogeneous Haldane model with PMF

**Figure 2.**
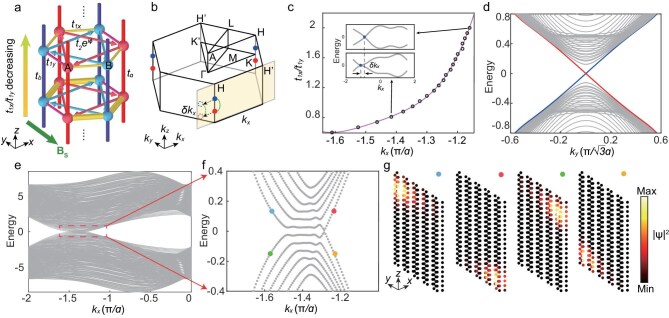
Inhomogeneous 3D Haldane model with PMF. (a) Schematic of the inhomogeneous 3D Haldane model. The ratio of the NN couplings along the *x* and *y* directions (${t}_{1x}/{t}_{1y}$) decreases along the *z* direction, generating a PMF **B**_s_ (green arrow) along the negative *y* direction. (b) 3D Bulk BZ and the 010-surface BZ (yellow sheet). The red and blue dots represent the WPs with opposite topological charges. The dashed black arrows indicate the shift of the WPs along the −${k}_{x}$ direction to induce the PMF. (c) Simulated (black circles) and fitted (purple line) curve that illustrates the relation between the ratios of the NN couplings (${t}_{1x}/{t}_{1y}$) and the shifts of the WPs along the −${k}_{x}$ direction. The ratios of the NN couplings (${t}_{1x}/{t}_{1y}$) are elaborately designed to make the shifts of WPs ($\delta {k}_{x}$) vary uniformly along the *z* direction. The insets show the bulk band dispersions along the ${k}_{x}$ direction with fixed ${k}_{y}$ = 0 and ${k}_{z} = - 0.78\pi /h$, demonstrating the shift of WPs (blue dots) when ${t}_{1x}/{t}_{1y}$ decreases from 2 (upper inset) to 0.8 (lower inset). (d) The projected band dispersions along the ${k}_{y}$ direction with fixed ${k}_{x} = - 1.33\pi /a$. The red and blue lines represent the chiral Landau levels corresponding to the two WPs with opposite topological charges. (e) The projected band dispersions of a finite inhomogeneous 3D Haldane model along the ${k}_{x}$ direction with open boundary conditions along both the y and z directions. (f) The zoomed projected band dispersion of the Landau levels (gray dotted lines). (g) The calculated eigenenergy distributions of the one-sided chiral hinge states of Fermi arcs at two different energies corresponding to two pairs of colored dots in (f).

We start with a 3D Haldane model [53] formed by stacking 2D Haldane models with interlayer couplings ${t}_a$ (red rods) and ${t}_b$ (blue rods) (see details in Note S1), as schematically shown in Fig. 2a. The sublattice sites A (red spheres) and B (blue spheres) have opposite on-site energies ± M and are coupled with nearest-neighbor real couplings ${t}_{1x}$ (yellow rods) and ${t}_{1y}$ (purple rods) along the *x* and *y* directions, respectively. The next-nearest-neighbor complex couplings ${t}_2{e}^{i\phi}$ (blue and red arrows) are introduced to break the TRS symmetry. When ${t}_{1x} = {t}_{1y}\ $and ${t}_a{t}_b < 0,$ the 3D Haldane model exhibits the simplest form of magnetic Weyl semimetal phase that hosts a single pair of WPs with opposite topological charges at ${K}_ \pm = ( {4\pi /3a,0, \pm {k}_w/h} )$ (blue and red dots) due to the TRS breaking, where ${k}_w = [ {M/( {{t}_b - {t}_a} )} ]$, as shown in Fig. 2b. The projection of the single pair of WPs on the surface BZ is connected by a single open surface Fermi arc (green arc).

We now construct the PMF by introducing an inhomogeneous modification to the 3D Haldane model and shifting the positions of the WPs. When ${t}_{1x}\ne{t}_{1y}$ and ${t}_{1x}/{t}_{1y}$ decreases along the *z* direction (yellow arrow in Fig. 2a), the single pair of WPs and the surface Fermi arcs move along the $- {k}_x$ direction in the surface BZ, as shown in Fig. 2b. The inset of Fig. 2c shows the bulk band dispersions along ${k}_x$ direction with fixed ${k}_y = {\mathrm{\ }}0$ and ${k}_z = {\mathrm{\ }} - 0.78\pi /{\mathrm{h}}$, demonstrating that the WPs (blue dots) shift $- \delta {k}_x$ when ${t}_{1x}/{t}_{1y}\ $ratios decrease from 2 to 0.8. By introducing layer-dependent tuning of ${t}_{1x}/{t}_{1y}$ ratios via the fitting curve (purple line) shown in Fig. 2c, the WPs shift uniformly among layers, which mimics the effect of a vector potential ${{\bf A}} = \delta {{\bf K}} = ( {\delta k( z ),0,\ 0} )$, generating a PMF ${{{\bf B}}}_{{\bf s}} = \nabla \times {{\bf A}} = - \frac{\rm d}{{\rm {d}\it z}}[ {\delta k( z )} ]{{\boldsymbol{e}}}_y$ (green arrow in Fig. 2a), where ${{\boldsymbol{e}}}_y$ is the unit vector along the *y* direction. This in-plane PMF drives the bulk states into Landau levels, where the chiral Landau levels (red and blue lines) at each WP exhibit opposite group velocities, as shown in Fig. 2d.

Besides the chiral bulk Landau levels along the ${k}_y$ direction, the PMF also turns surface Fermi arcs into Landau plateaus along the ${k}_x$ direction and deforms them into one-sided chiral hinge states of Fermi arcs at the diagonal boundaries of the front and back surfaces. To demonstrate this exotic phenomenon, we construct a rectangular structure which is finite in the *y*-*z* plane and periodic along the *x* direction, and calculate its dispersion along the ${k}_x$ direction, as shown in Fig. 2e. The Landau levels of surface Fermi arcs under PMF can be clearly observed in Fig. 2f, which deform at the boundaries of the front and back surfaces and generate one-sided chiral hinge states (colored dots) localized at one edge of the front surface and the opposite edge of the back surface. Figure 2g shows the unique spatial distributions of the one-sided chiral hinge states marked by two pairs of colored dots in Fig. 2f, from which we can see that, at a specific energy, the one-sided chiral hinge states are localized at a pair of diagonal hinges and propagate unidirectionally along opposite directions. When we change the energy or reverse the PMF, the chiral hinge states of Fermi arcs can be switched between the two pairs of diagonal hinges. Note that the one-sided chiral hinge states of Fermi arcs originate from the surface arc states under PMF, which is totally different from that (bulk axion angle $\theta = \pi $ and gapped surfaces possessing surface Chern numbers of *C*_s_ = ±1/2) of the chiral hinge states in photonic axion insulators [67–71].

### Photonic realization of 3D QHE of Fermi arcs in an inhomogeneous magnetic Weyl photonic crystal

**Figure 3.**
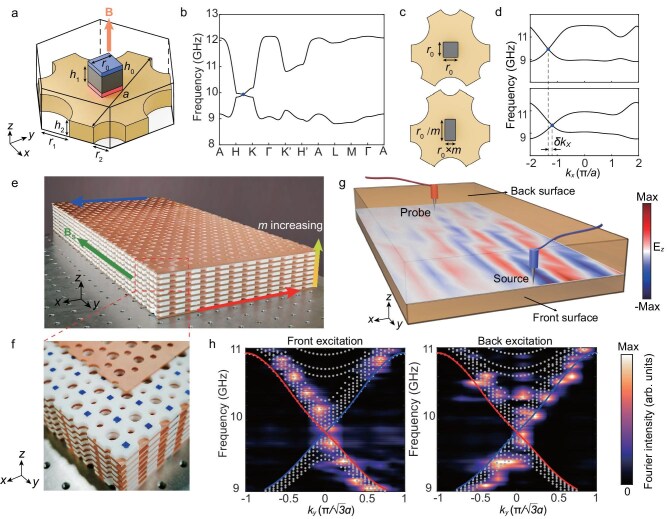
Photonic realization of 3D QHE of Fermi arcs in an inhomogeneous magnetic Weyl photonic crystal. (a) Unit cell of a 3D gyromagnetic photonic crystal that consists of a square YIG rod (dark gray color) sandwiched between two permanent magnets (blue and red colors, providing a magnetic field *B* = 110 mT) standing on a perforated metallic plate (yellow color). The geometrical parameters are radii $r_1 = 4.6{\mathrm{\ mm\ }}$and $r_2 = 2.8{\mathrm{\ mm}}$, in-plane lattice constant $a = 20\ {\mathrm{mm}}$, heights $h_1 = 3.0\ {\mathrm{mm}},\ h_2 = 2.0\ {\mathrm{mm}},\ h_0\ = 0.75\ {\mathrm{mm}}$, and side length $r_0 = 3.8\ {\mathrm{mm}}$. (b) Simulated bulk band structure of the 3D gyromagnetic photonic crystal with a single pair of WPs (blue dot) at 9.9 GHz. (c and d) Layer-dependent unit cells (length = ${r}_0/m$, width =$\ {r}_0\times m$, *m* < 1) and their corresponding bulk band dispersions along the ${k}_{x}$ direction. The WPs (blue dots) shift $\delta {k}_{x}$ when *m* decreases from 1 (upper panel) to 0.7 (lower panel). (e) Photograph of the fabricated experimental sample with 8 layers along the *z* direction and 12 × 14 unit cells in the *x-y* plane, with *m* increasing along the *z* direction (yellow arrow) and inducing a PMF whose *y* component (green arrow) is perpendicular to the surface Fermi arcs. The red and blue arrows represent the one-sided chiral hinge states of Fermi arcs. (f) Close-up view of the sample. The first layer of the copper plate has been shifted to see the inner structure. The YIG rods sandwiched between two magnets (blue square) are embedded in the air foam (white color) to fix their positions. (g) Schematic of the experimental setup. The electric field distribution of the bulk state is measured using a pair of source and probe antennas (blue and red colors) inserted into the bulk. (h) Measured (color map) and simulated (blue and red lines) chiral Landau levels along the ${k}_{y}$ direction with front (left panel) and back (right panel) excitations, respectively, where ${k}_{x} = - 1.27\pi /\alpha $.

Now we implement the inhomogeneous 3D Haldane model in a 3D gyromagnetic photonic crystal with TRS breaking, in which the designed unit cell is composed of a square yttrium iron garnet (YIG) rod (dark gray color), sandwiched between and biased by one pair of permanent magnets (red and blue color) standing on a perforated metallic plate (yellow color), as illustrated in Fig. 3a, with geometrical parameters, $h_1 = 3.0\ {\mathrm{mm}},\ h_2 = 2.0\ {\mathrm{mm}},\ h_0\ = 0.75\ {\mathrm{mm}},r_0 = 3.8\ {\mathrm{mm}}$, and $a = 20\ {\mathrm{mm}}$. The permanent magnets provide a magnetic field of *B* = 110 mT (brown arrow) to magnetize the YIG rods to break the TRS, and the air holes ($r_1 = 4.6{\mathrm{\ mm}}$ and $r_2 = 2.8{\mathrm{\ mm}}$) positioned at the two sets of inequivalent corners of the hexagonal unit cell enable interlayer couplings and in-plane inversion symmetry breaking simultaneously (see details in Methods and Note S2). Figure 3b shows the simulated bulk band structure of the 3D gyromagnetic photonic crystal, in which two bands linearly intersect at momenta $( {{k}_x,{\mathrm{\ }}{k}_y,{\mathrm{\ }}{k}_z} ) = ( { - 1.332\pi /a,0, \pm 0.530\pi /h} )$ with $h = {h}_1 + {h}_2 + 2{h}_0$ along the high-symmetry HK line in the 3D BZ, forming a single pair of WPs (blue dot) at 9.9 GHz. We then deform the square YIG rods and permanent magnets into rectangles (length = ${r}_0/m$, width =$\ {r}_0\times m$, and *m* < 1) to introduce the layer-dependent ${t}_{1x}/{t}_{1y}$ ratios to shift the WPs and construct the PMF. Figure 3c and d show the top view of two unite cells with different YIG rods (*m* = 1 and 0.7) and their corresponding bulk band dispersions along the ${k}_x$ direction, from which we can see the WPs (blue dots) shift $\delta {k}_x$ as *m* decreases from 1 to 0.7, generating a uniform PMF whose *y*-component is normal to the surface Fermi arcs, which quantizes the bulk and surface arc states (see details about the surface arc states in Note S3) to Landau level plateaus. Note that in real gyromagnetic photonic crystals, deforming square YIG rods into rectangular ones also alters the interlayer couplings and induces an additional shift of the WPs along the ${k}_z\ $direction. To establish a more accurate and rigorous mapping between the theoretical framework and gyromagnetic photonic crystals, we developed a refined TB model that incorporates layer-dependent interlayer couplings (see details in Note S1). The results obtained from this refined TB model show excellent agreement with those from the real gyromagnetic photonic crystals. To implement the 3D inhomogeneous Haldane model in a 3D gyromagnetic photonic crystal, we have elaborately designed eight distinct unit cells with different parameters *m* to control the shifts of WPs along the ${k}_x$ direction, as shown in Table 1. By arranging these different unit cells along the *z* direction with *m* increasing (yellow arrow in Fig. 3e), we design an inhomogeneous 3D magnetic Weyl photonic crystal that implements the inhomogeneous 3D Haldane model shown in Fig. 2a. Figure 3e and f present the photograph of the fabricated experimental sample, which comprises eight layers along the *z* direction and 12 × 14 unit cells in the *x-y* plane. The YIG rods sandwiched between two permanent magnets (blue squares) are embedded in the air foam (white color) to fix their positions. The geometric parameter *m* increases along the *z* direction (yellow arrow), resulting in the WPs shifting toward the ${k}_x$ direction and generating a PMF with a uniform *y*-component (green arrow). To characterize the chiral Landau levels induced by the PMF, we place a source antenna (blue color) on the back or front (010) surfaces to excite the chiral bulk Landau modes and use a probe antenna (red color) to map their electric field distributions in the *x-y* plane, as shown in Fig. 3g. Then we perform Fourier transformations to the measured electric field distributions of the chiral Landau modes to obtain their dispersion relations, as shown in Fig. 3h, from which we can observe that the front (back) surface excitation excites the chiral bulk Landau modes propagate along $- {k}_y$ (${k}_y$) direction with negative (positive) group velocity, and the measured results (color maps) match well with the simulation results (red and blue lines). These results confirm that the PMF induces chiral Landau levels with opposite group velocities corresponding to oppositely charged WPs.

**Table 1.** Values of the geometrical parameters of the 3D inhomogeneous magnetic Weyl photonic crystal and locations of the WPs for each layer (from bottom to top).

Index of the layers	*m*	Length (mm)	Width (mm)	Position of the WPs
Layer 1	0.687	5.5	2.6	$( { - 1.180\pi /a,0, \pm 0.695\pi /h} )$
Layer 2	0.714	5.3	2.7	$( { - 1.202\pi /a,0, \pm 0.657\pi /h} )$
Layer 3	0.746	5.1	2.8	$( { - 1.223\pi /a,0, \pm 0.617\pi /h} )$
Layer 4	0.783	4.8	3.0	$( { - 1.245\pi /a,0, \pm 0.588\pi /h} )$
Layer 5	0.825	4.6	3.1	$( { - 1.267\pi /a,0, \pm 0.562\pi /h} )$
Layer 6	0.873	4.4	3.3	$( { - 1.289\pi /a,0, \pm 0.543\pi /h} )$
Layer 7	0.931	4.1	3.5	$( { - 1.310\pi /a,0, \pm 0.535\pi /h} )$
Layer 8	1.000	3.8	3.8	$( { - 1.332\pi /a,0, \pm 0.530\pi /h} )$

Considering manufacturing precision requirements, the length and width of the YIG rods are recorded to 1 decimal place (0.1 mm).

### Observation of one-sided chiral hinge states of Fermi arcs

**Figure 4.**
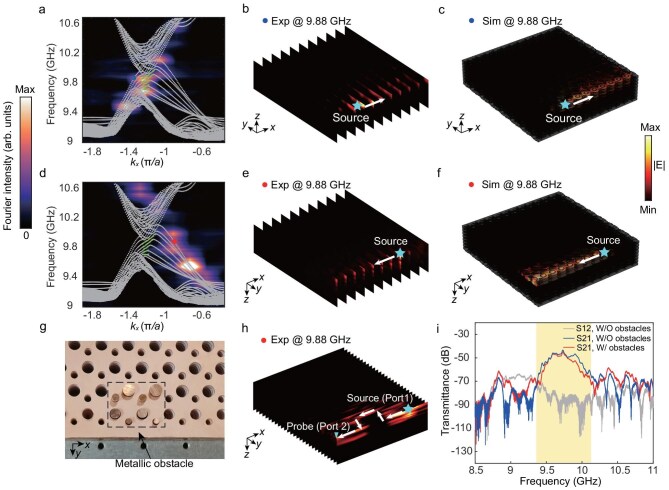
Observation of one-sided chiral hinge states of Fermi arcs. (a) Measured (color map) and simulated (gray dotted line) band dispersions of the one-sided chiral hinge states of Fermi arcs of the 3D inhomogeneous magnetic Weyl photonic crystal along the *k_x_* direction. The green dashed line indicates the Landau levels, and the blue dot represents the chiral hinge states of Fermi arcs. (b and c) Measured (b) and simulated (c) electric field distribution of the chiral hinge states of Fermi arcs at 9.88 GHz for ${k}_{x} = - 1.32\pi /a\ $(blue dot in a). The cyan star represents the point source. (d–f) Similar to (a–c) but for the chiral hinge states at 9.88 GHz with ${k}_{x} = - 0.93\pi /a\ $(red dot in d) at the diagonal hinge. (g) Photograph of copper pillars inserted into the sample as metallic obstacles (black dashed square). (h) Measured electric field distribution of the chiral hinge states of Fermi arcs circumventing the metallic obstacles. (i) Measured rightward (gray line, S12) and leftward (blue line, S21) transmission spectra without metallic obstacles, and the leftward transmission spectrum with metallic obstacles (red line, S21).

Finally, we experimentally demonstrate the one-sided chiral hinge states of Fermi arcs in the inhomogeneous magnetic Weyl photonic crystal, a hallmark of the 3D QHE of Fermi arcs that has never been observed experimentally in any physical system. Figure 4a and d show the simulated ${k}_x$-projected dispersion of a finite rectangular supercell structure (gray dotted lines) with open boundary conditions in the *y* and *z* directions and periodic boundary conditions in the *x* direction, in which we can see a sequence of Landau levels (green dashed lines) emerges in the frequency range of 9.76–9.9 GHz. These Landau levels exhibit slight tilting rather than perfect flatness due to the interlayer boundary energy variations (see details in Note S4). More interestingly, the Landau plateaus deform near the surface boundaries and generate one-sided chiral hinge states of Fermi arcs (red and blue dots) at one boundary of the front surface and the diagonal boundary of the back surface (see Note S5). Note that, unlike the TB model prediction in Fig. 2f and g, the Landau levels and chiral hinge states of Fermi arcs at frequencies exceeding the Weyl frequency merge with the bulk bands and cannot be distinguished in the gyromagnetic Weyl photonic crystals. Consequently, we only focus on the lower half parts of the Landau levels and chiral hinge states of Fermi arcs. To directly observe the one-sided chiral hinge states of Fermi arcs, we put a point source (blue stars) at the two diagonal hinges and use another probe antenna to measure their electric field distributions at 9.88 GHz, as shown in Fig. 4b and e, the chiral hinge states of Fermi arcs are tightly confined and propagate unidirectionally along the two diagonal hinges, matching well with the simulation results shown in Fig. 4c and f (see the propagation behaviors of one-sided chiral hinge states on the *y-z* surface in Note S6). Then we perform Fourier transforms of the measured electric field distributions of the chiral hinge states to retrieve their dispersion relations (color maps), which match well with the simulation results (gray dotted lines), as shown in Fig. 4a and d. More interestingly, the chiral hinge states of Fermi arcs can be switched between two pairs of diagonal hinges by reversing the PMF (see Note S7). To examine the topologically protected robustness of the chiral hinge states of Fermi arcs, as shown in Fig. 4g, we insert a metallic obstacle (black dashed square) in the path of the chiral hinge states. Figure 4h shows the measured electric-field distribution of the chiral hinge states, from which we observe that they circumvent the obstacle smoothly without backscattering. Figure 4i shows the measured transmission spectra of the S21 (blue line) and S12 (gray line) without an obstacle, and S21 with an obstacle (red line). The high contrast between the leftward (S21) and rightward (S12) transmissions in the yellow region demonstrates the unidirectionality and nonreciprocity of the chiral hinge states of Fermi arcs, and the almost overlapped leftward transmission spectra (S21) with (red line) and without (blue line) obstacle further verify the robustness of the chiral hinge states of Fermi arcs.

## DISCUSSION

In summary, we have experimentally demonstrated the chiral Landau levels and one-sided chiral hinge states of Fermi arcs in an inhomogeneous magnetic Weyl photonic crystal, constituting the conclusive experimental evidence for the first photonic realization of the exotic 3D QHE of Fermi arcs. The magnetic Weyl photonic crystal with PMF serves as an ideal platform for exploring 3D quantum Hall physics with TRS breaking. In the presence of PMF, the bulk states and the closed Fermi loop consisting of Fermi arcs and WPs give rise to chiral Landau levels and one-sided chiral hinge states, which are the hallmarks of 3D QHE of Fermi arcs. Moreover, we demonstrate that the one-sided chiral hinge states of Fermi arcs can be switched between two pairs of diagonal hinges by reversing the PMF. Our work not only provides an ideal platform for exploring 3D quantum Hall physics but also opens new avenues for the design of robust photonic devices. We envision that other classical analogs of QHEs, such as the 3D quantum anomalous Hall effect in Weyl semimetals [72] and four-dimensional QHE [73–76], can also be experimentally realized in 3D gyromagnetic photonic crystals.

## METHODS

### Numerical simulation

All numerical results presented in this work are simulated using the RF module of COMSOL Multiphysics. Both the copper plates and permanent magnets are modeled as perfect electric conductors (PECs). The bulk band structure is simulated using a hexagonal unit cell with periodic boundary conditions in all three directions. The projected dispersions along the ${k}_x$ direction were calculated using a 1 × 14 × 8 supercell with periodic boundary conditions along the *x* direction, and the other boundaries were set as PEC boundary conditions. The relative permeability tensor of the gyromagnetic materials (YIG rods) has the form


(1) \begin{eqnarray*}
{\boldsymbol{\mu }} = \left( {\begin{array}{@{}*{3}{c}@{}} {{\mu }_r}&{j\kappa }&0\\ { - j\kappa }&{{\mu }_r}&0\\ 0&0&1 \end{array}} \right),
\end{eqnarray*}

where ${\mu }_r = 1 + {\omega }_m( {{\omega }_0 + i\alpha \omega } )/( {{{( {{\omega }_0 + i\alpha \omega } )}}^2 -} {\omega }^2 ),$  $\kappa = {\omega }_m{\omega }_0/( {{{( {{\omega }_0 + i\alpha \omega } )}}^2 - {\omega }^2} )$, ${\omega }_m = \gamma {\mu }_0{M}_s$, ${\omega }_0 = \gamma {\mu }_0{H}_0$, ${\mu }_0{H}_0$ is the external magnetic field along the *z* direction. $\gamma = 1.76 \times {10}^{11}{\mathrm{\ }}{{\mathrm{s}}}^{ - 1}{\mathrm{\ }}{{\mathrm{T}}}^{ - 1}$ is the gyromagnetic ratio, $\alpha = 0.0088$ is the damping coefficient, and *ω* is the operating frequency. Under a 110 mT external magnetic field, Fig. S1a and b display the frequency dispersions of the permeability tensor components ${\mu }_r$ and $\kappa$, respectively. The Weyl frequency is far away from the gyromagnetic material’s resonance frequency, resulting in negligible material dispersion across the operating frequency range.

### Sample fabrication

In the experiment, we adopt commercially available gyromagnetic materials (YIG ferrite) and permanent magnets to break the TRS. The YIG exhibits a saturation magnetization ${M}_{\mathrm{s}} = 1780{\mathrm{\ Gauss}}$. Their relative permittivity (${\varepsilon }_r = 14.3 + 0.003{\mathrm{i}}$) and permeability (${\mu }_r \approx 1$) remain nearly constant across microwave frequencies. The permanent magnets (Sm_2_Co_17_) are nickel-electroplated with a 2-μm-thick coating. A pair of magnets generates a uniform 110 mT external magnetic field to magnetize the gyromagnetic rods. The copper plates are fabricated by depositing a 0.035 mm-thick copper layer onto a Teflon woven-glass fabric laminate substrate and perforating the plates with air holes using a laser-cutting technique. We use perforated dielectric foam (ROHACELL 31HF) with a relative permittivity of 1.04 and a loss tangent of 0.0025 to fix the sandwiched YIG rods and permanent magnets.

### Experimental setups

In the experimental measurements, we adopt two microwave dipole antennas connected to a vector network analyzer (Keysight E5080) as the source and probe. By inserting the probe antenna into the air holes one by one and scanning along the *z*-direction in small steps, we can map the complex electric field distributions within the bulk or on the hinges of the experimental sample. To measure the projected dispersion relations, the source antenna is positioned either on the surfaces to excite the chiral bulk Landau modes or at the hinges to excite the chiral hinge states of Fermi arcs. The probe antenna then scans to map the corresponding complex electric field distributions (in the *x-y* plane for the zeroth bulk modes or along the hinges for the chiral hinge states). For the chiral bulk Landau modes, we perform a 2D Fourier transformation at each frequency with a fixed ${k}_x = - 1.27\pi /a$ to plot the chiral Landau levels along the ${k}_y$ direction. While for the chiral hinge states, we perform a 1D Fourier transformation to obtain their projected dispersions along the ${k}_x$ direction.

## Supplementary Material

nwag251_Supplemental_File
