# Supplementary material for: Three-dimensional photonic quantum Hall effect of Fermi arcs
Source: Natl Sci Rev. 2026 Apr 29;13(13):nwag251. doi: 10.1093/nsr/nwag251 (PMC13343493; doi:10.1093/nsr/nwag251)
Supplement: nwag251_Supplemental_File [file nwag251_supplemental_file.docx]

**Supplementary Information of**

**“Three-dimensional photonic quantum Hall effect of Fermi arcs”**

Zhengting Wu^1, †^, Minqi Cheng^1, †^, Ziyao Wang^1^, Siqi Xu^1^, Jingming Chen^1^, Yan Meng^2^, Xiang Xi^2^, Xiankai Sun^3^, Perry Ping Shum^1^, Haizhou Lu^4,5^, Zhen Gao^1, *^

^1^State Key Laboratory of Optical Fiber and Cable Manufacturing Technology, Department of Electronic and Electrical Engineering, Guangdong Key Laboratory of Integrated Optoelectronics Intellisense, Southern University of Science and Technology, Shenzhen 518055, China

^2^School of Electrical Engineering and Intelligentization, Dongguan University of Technology, Dongguan 523808, China.

^3^Department of Electronic Engineering, The Chinese University of Hong Kong, Hong Kong 999077, China.

^4^State Key Laboratory of Quantum Functional Materials, Department of Physics, and Guangdong Basic Research Center of Excellence for Quantum Science, Southern University of Science and Technology (SUSTech), Shenzhen 518055, China

^5^Quantum Science Center of Guangdong-Hong Kong-Macao Greater Bay Area (Guangdong), Shenzhen 518045, China

^*^Corresponding author: gaoz@sustech.edu.cn

^†^Equally contributed to this work

**Contents**

**Note 1. The tight-binding (TB) Hamiltonian of the inhomogeneous three-dimensional (3D) Haldane model without and with time-reversal symmetry (TRS) breaking**

**Note 2. Modeling magnetic Weyl photonic crystal in COMSOL Multiphysics**

**Note 3. Fermi arc surface states of the magnetic Weyl photonic crystal**

**Note 4. Landau Level formation and boundary modification**

**Note 5. Simulated eigen-field distributions of the one-sided chiral hinge states of Fermi arcs**

**Note 6. Propagation behaviors of the one-sided chiral hinge states on the *y-z* surface**

**Note 7. Switching the one-sided chiral hinge states of Fermi arcs by reversing the PMF**

**Note 1. The tight-binding (TB) Hamiltonian of the inhomogeneous three-dimensional (3D) Haldane model without and with time-reversal symmetry (TRS) breaking**

**(1) The TB results of the inhomogeneous 3D Haldane model with TRS**

To demonstrate the significance of the TRS breaking in the photonic realization of the 3D quantum Hall effect (QHEF) of Fermi arcs, we present the TB results of the 3D inhomogeneous Haldane model with TRS (i.e., $t_{2}=0$ or $\phi=0$) that supports nonchiral topological hinge states of Fermi arcs in 3D acoustic Weyl crystals [Ref. 64 in the main text] and synthetic dimensions [Ref. 65 and 66 in the main text].

Fig. S1a displays the bulk band structure of the inhomogeneous 3D Haldane model (Fig. 2a in the main text) with TRS (i.e., $t_{2}=0$ or $\phi=0$) along the high-symmetry lines for onsite energies $M=0.5$ and $M=3$, respectively. The blue and red dots denote Weyl points (WPs) with opposite topological charges on the HK and H′K′ lines. By varying the onsite energy, these WPs shift along the $k_{z}$ direction. Since the system preserves TRS, it hosts two pairs of WPs. The Fermi arcs (green line in Fig. S1b) connecting these WPs exhibit identical movement trajectories. Unlike the inhomogeneous 3D Haldane model with TRS breaking in our work, the Fermi arcs of the inhomogeneous 3D Haldane model with TRS are oriented along the $k_{x}$ direction rather than the $k_{z}$ direction, as illustrated in Fig. S1b. Consequently, the movement of the WPs and Fermi arcs is perpendicular to the Fermi arc orientation (along $k_{z}$ direction).


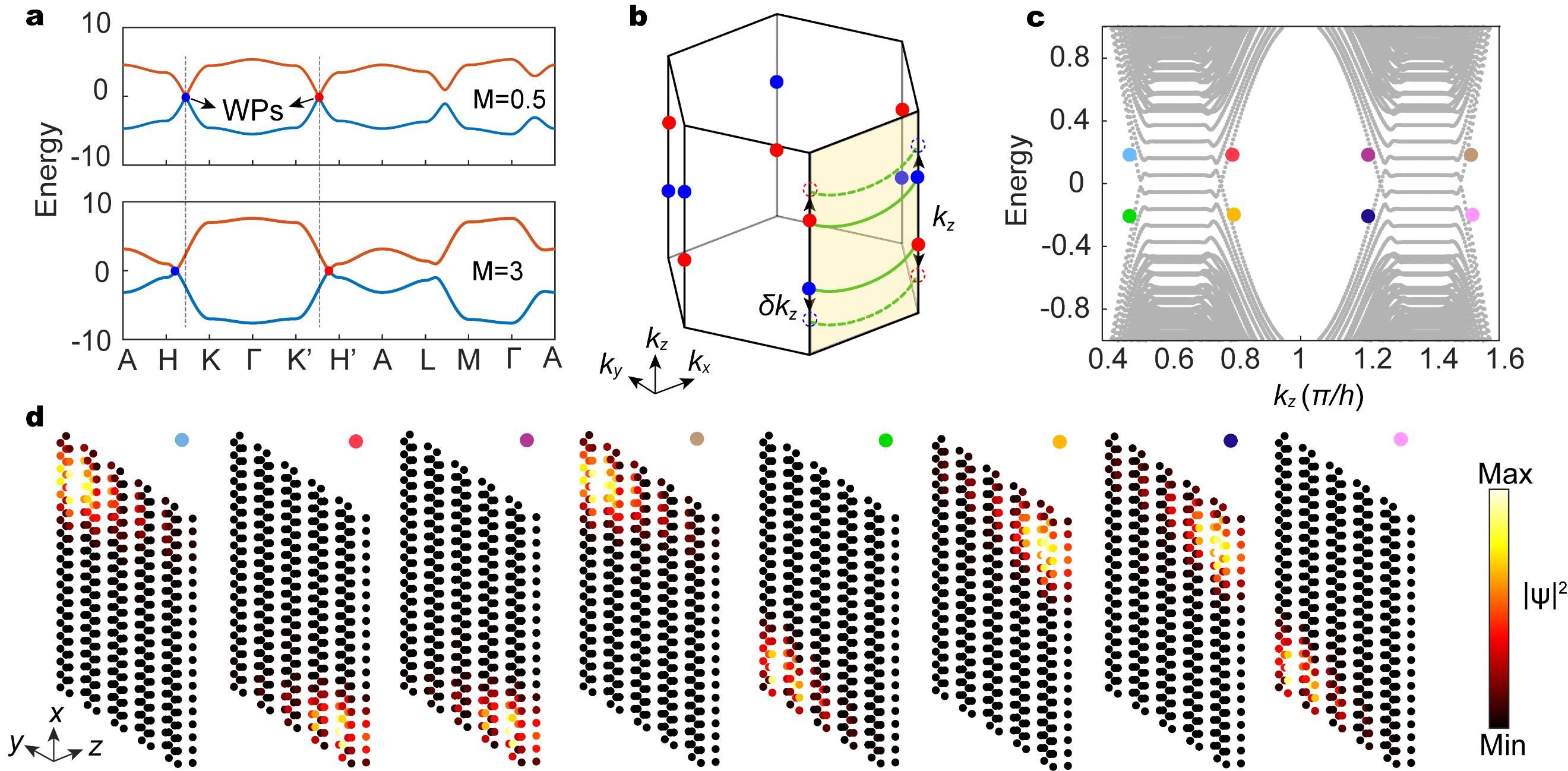


**Fig. S1 | TB results of the inhomogeneous 3D Haldane model with TRS. a** The bulk band structure of the inhomogeneous 3D Haldane model with TRS, with the onsite energy $M=0.5$ (upper panel) and $M=3$ (lower panel), respectively. Other TB parameters are$t_{1x}=t_{1y}=1, t_{2}=0, t_{a}=-t_{b}=2, \mathrm{and} \phi=0$. **b** 3D Bulk Brillouin zone (BZ) and the projected (010) surface BZ (yellow sheet). The red and blue dots represent the WPs with opposite topological charges. The black arrows indicate the shift of the WPs along the $k_{z}$ direction to induce the PMF. **c** The projected band dispersions of a supercell of the inhomogeneous 3D Haldane model with TRS along the $k_{z}$ direction, with open boundary conditions along the *x* and *y* directions and periodic boundary conditions along the *z* direction. **d** The calculated eigenenergy distributions of the hinge states of Fermi arcs at two different energies corresponding to four pairs of colored dots in **c**.

We then construct a rectangular structure that is finite in the *x*-*y* plane and periodic along the z-axis. The dispersion along $k_{z}$ direction is calculated, as shown in Fig. S1c. In contrast to our TRS-breaking system, the dispersion of the inhomogeneous 3D Haldane model with TRS is symmetric along the $k_{z}$ direction. Fig. S1d shows the spatial distributions of hinge states of Fermi arcs of the 3D inhomogeneous Haldane model with TRS, corresponding to the four pairs of colored dots in Fig. S1c, where the Landau levels deform at the boundaries and form hinge states of Fermi arcs. At a specific energy, we can see that the hinge states of Fermi arcs on a pair of diagonal hinges propagate bidirectionally, indicating that the hinge states of Fermi arcs are non-chiral in the TRS-preserving system.

1. **The TB Bulk Hamiltonian of the inhomogeneous 3D Haldane model with TRS breaking**

The schematic of the inhomogeneous 3D Haldane model with TRS breaking is shown in Fig. 2a of the main text, which is constructed by stacking the 2D Haldane model with interlayer couplings $t_{a}$ and $t_{b}$. The Hamiltonian $H_{3Din-hldn}\left( k \right)$ is a 2-by-2 matrix:

$$H_{3Din-hldn, 11}\left( k_{1}, k_{2},k_{3} \right)=+M+\left( t_{2}e^{i\phi}e^{ik_{1}}+t_{2}e^{i\phi}e^{-ik_{2}}+t_{2}e^{-i\phi}e^{ik_{1}-ik_{2}}+t_{a}e^{ik_{3}}+h.c. \right) (S1a)$$

$$H_{3Din-hldn, 22}\left( k_{1}, k_{2},k_{3} \right)=-M+\left( t_{2}e^{-i\phi}e^{ik_{1}}+t_{2}e^{-i\phi}e^{-ik_{2}}+t_{2}e^{i\phi}e^{ik_{1}-ik_{2}}+t_{b}e^{ik_{3}}+h.c. \right) (S1b)$$

$$H_{3Din-hldn, 12}\left( k_{1}, k_{2},k_{3} \right)=t_{1y}+t_{1x}{(e}^{-ik_{1}}+e^{-ik_{2}}) (S1c)$$

$$H_{3Din-hldn, 22}\left( k_{1}, k_{2},k_{3} \right)=t_{1y}+t_{1x}(e^{ik_{1}}+e^{ik_{2}}) (S1d)$$

in which *h.c.* represents the complex conjugation, and $k_{i}=\boldsymbol{k\cdot}\boldsymbol{a}_{\boldsymbol{i}} \in[0, 2\pi] (i = 1, 2, 3)$ denote the reduced wave numbers, with the primitive lattice vectors $\boldsymbol{a}_{\boldsymbol{1}}\boldsymbol{=}\left( \frac{1}{2},\frac{\sqrt{3}}{2},0 \right), \boldsymbol{a}_{\boldsymbol{2}}\boldsymbol{=}\left( -\frac{1}{2},\frac{\sqrt{3}}{2},0 \right),\boldsymbol{a}_{\boldsymbol{3}}\boldsymbol{=}(0,0,1)$, and the wave vector $\boldsymbol{k=}(k_{x},k_{y},k_{z})$.

The bulk band structure of the inhomogeneous 3D Haldane model with TB parameters$t_{1x}=t_{1y}=1, t_{2}=-0.5, t_{a}=-t_{b}=1, \phi=\frac{\pi}{2}$, and $M=4$along the high-symmetry lines in the momentum space is plotted in Fig. S2a, which only supports a single pair of WPs (blue dot). Figs. S2b-S2c shows the projected bulk band structures along the $k_{x}$ direction with $t_{1x}=0.8$ (Fig. S2b) and $t_{1x}=2$ (Fig. S2c), respectively, demonstrating the shift of WPs under different $t_{1x}/t_{1y}$ratios. Consequently, by sweeping the $t_{1x}/t_{1y}$ratios and getting the corresponding $k_{x}$ coordinates of the WPs, we can plot the fitted curve that illustrates the relation between the $t_{1x}/t_{1y}$ ratios and the shifts of the WPs in Fig. 2c of the main text.


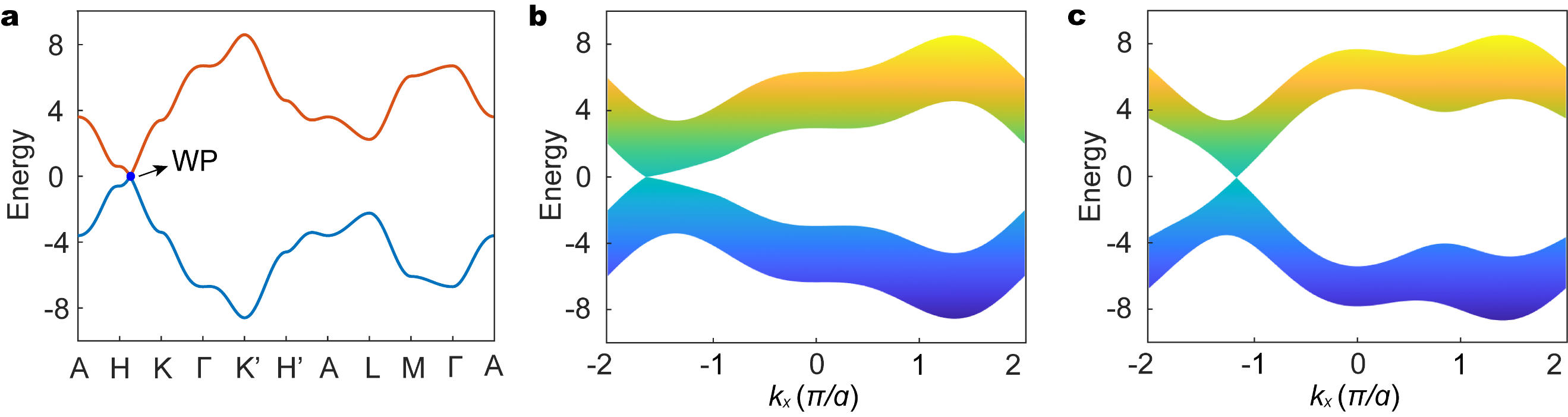


**Fig. S2 | TB bulk band structures of the inhomogeneous 3D Haldane model with TRS breaking. a** The bulk band structure of the inhomogeneous 3D Haldane model with TRS breaking along the high-symmetry path, the blue dot represents the WP. **b, c** The projected bulk band structures of the inhomogeneous 3D Haldane model with TRS breaking along the $k_{x}$ direction with $t_{1x}=0.8$ (**b**) and $t_{1x}=2$ (**c**), respectively.

**(3) The TB supercell Hamiltonian**

To study the chiral zeroth Landau levels of the inhomogeneous 3D Haldane model with TRS breaking, we calculate the projected dispersions of a finite supercell consisting of N layers (grey area) with open boundary conditions along the *z-*direction and periodic boundary conditions along the *x-* and *y-*directions. The top view of the supercell is shown in Fig. S3a, which contains 4$\times$N sites. To make the WPs shift uniformly alone the $k_{x}$ direction, the nearest-neighbor (NN) couplings alone the *x* direction $t_{1x}$ is layer-dependent ($t_{1x}\left( l_{z} \right), l_{z}= 1, 2, 3,\ldots,N, are the indexs of layers)$. The Hamiltonian $(H_{3Din-hldn}^{slab-\left( 0,0,1 \right)})$ can be written as a 4$\times$N-by-4$\times$N matrix:

$$H_{3Din-hldn}^{slab-\left( 0,0,1 \right)}\left( k_{x},k_{y} \right)=\left[ \begin{matrix} H_{0}\left( k_{x}, k_{y} \right)\left. \right|_{l_{z}=1} & H_{couple} & \cdots& 0 & 0 \\ H_{couple}^{*} & H_{0}\left( k_{x}, k_{y} \right)\left. \right|_{l_{z}=2} & \cdots& 0 & 0 \\ 0 & H_{couple}^{*} & \cdots& 0 & 0 \\ \vdots& \vdots& \ddots& \vdots& \vdots\\ 0 & 0 & \cdots& H_{0}\left( k_{x}, k_{y} \right)\left. \right|_{l_{z}=N-1} & H_{couple} \\ 0 & 0 & \cdots& H_{couple}^{*} & H_{0}{\left( k_{x}, k_{y} \right)|}_{l_{z}=N} \end{matrix} \right] (S2)$$

Where $H_{0}$ and $H_{couple}$ are 4-by-4 matrices:

$$H_{0, 11}\left( k_{x}, k_{y} \right)=H_{0, 33}\left( k_{x}, k_{y} \right)=+M+t_{2}e^{i\phi}e^{ik_{x}}+t_{2}e^{-i\phi}e^{-ik_{x}} (S3a)$$

$$H_{0, 22}\left( k_{x}, k_{y} \right)=H_{0, 44}\left( k_{x}, k_{y} \right)=-M+t_{2}e^{-i\phi}e^{ik_{x}}+t_{2}e^{i\phi}e^{-ik_{x}} (S3b)$$

$$H_{0, 12}\left( k_{x}, k_{y} \right)=H_{0, 21}^{*}\left( k_{x}, k_{y} \right)=t_{1x}\left( l_{z} \right)+t_{1x}\left( l_{z} \right)e^{ik_{x}} (S3c)$$

$$H_{0, 13}\left( k_{x}, k_{y} \right)=H_{0, 31}^{*}\left( k_{x}, k_{y} \right)=t_{2}e^{i\phi}+t_{2}e^{-i\phi}e^{ik_{x}}+t_{2}e^{i\phi}e^{-ik_{y}}+t_{2}e^{i\phi}e^{i(k_{x}-k_{y})} (S3d)$$

$$H_{0, 14}\left( k_{x}, k_{y} \right)=H_{0, 41}^{*}\left( k_{x}, k_{y} \right)=t_{1y}e^{-ik_{y}} (S3e)$$

$$H_{0, 23}\left( k_{x}, k_{y} \right)=H_{0, 32}^{*}\left( k_{x}, k_{y} \right)=t_{1y} (S3f)$$

$$H_{0, 24}\left( k_{x}, k_{y} \right)=H_{0, 42}^{*}\left( k_{x}, k_{y} \right)=t_{2}e^{i\phi}+t_{2}e^{-i\phi}e^{-ik_{x}}+t_{2}e^{i\phi}e^{-ik_{y}}+t_{2}e^{-i\phi}e^{i({-k}_{x}-k_{y})} (S3g)$$

$$H_{0, 34}\left( k_{x}, k_{y} \right)=H_{0, 43}^{*}\left( k_{x}, k_{y} \right)=t_{1x}\left( l_{z} \right)+t_{1x}\left( l_{z} \right)e^{-ik_{x}} (S3h)$$

$$H_{couple}=\left[ \begin{matrix} t_{a} & 0 & 0 & 0 \\ 0 & t_{b} & 0 & 0 \\ 0 & 0 & t_{a} & 0 \\ 0 & 0 & 0 & t_{b} \end{matrix} \right] (S3i)$$

The projected band dispersions along the $k_{x}$ direction for $k_{y}=0$ are shown in Fig. S3b, in which we can observe a chiral zeroth Landau level plateau (red dot). The projected band dispersion along the $k_{y}$ direction is shown in Fig. 2d of the main text, which also shows the chiral zeroth Landau levels corresponding to the two WPs with opposite topological charges. The calculated eigenenergy distributions of the eigenstates (corresponding to the colored dots in Fig. S3b) are shown in Fig. S3c.


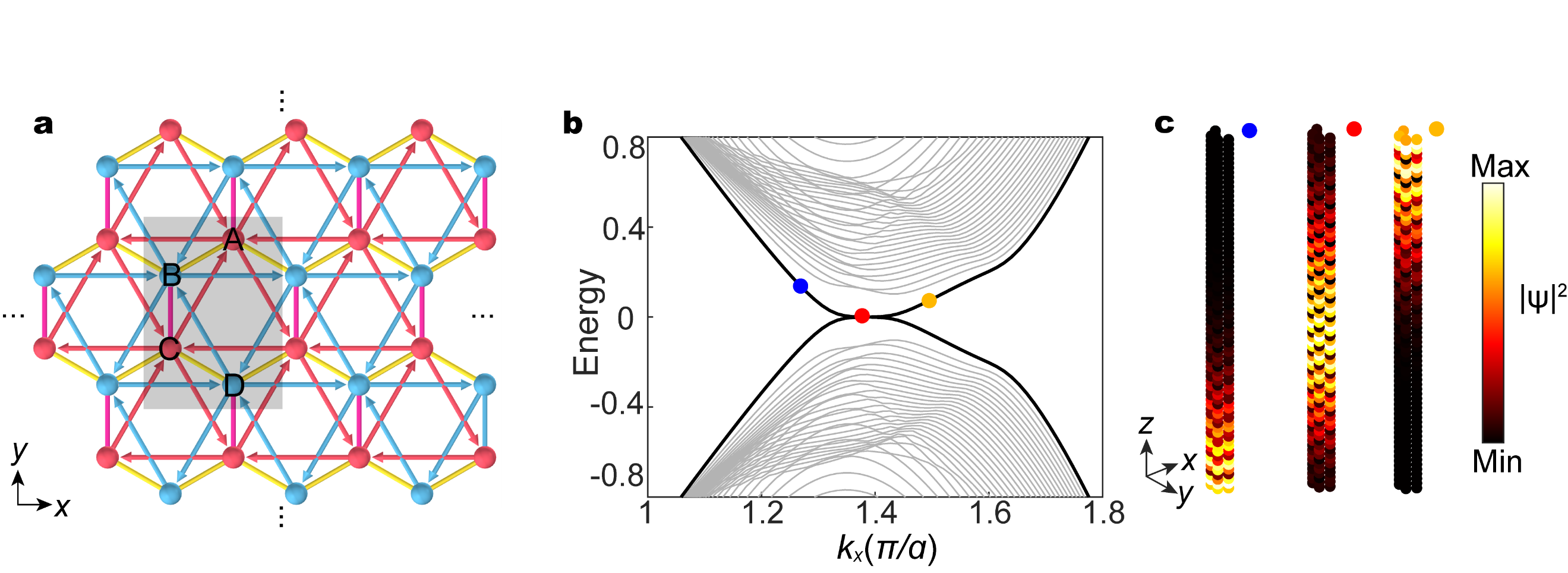


**Fig. S3 | a** Top view of the inhomogeneous 3D Haldane model with TRS breaking, whose primitive unit cell is shaded in gray. **b** The projected band dispersions of a finite inhomogeneous 3D Haldane model along the $k_{x}$ direction for $k_{y}=0$ with open boundary conditions along the *z* directions and periodic boundary conditions along the *x* and *y* directions. **c** The calculated eigenenergy distributions of the eigenmodes marked by the colored dots in **b**.

To study the chiral hinge states of Fermi arcs, we calculate the surface dispersions of a finite rectangular structure which consists of T$\times$N unit cells in the *y*-*z* plane and periodic along the *x* direction, whose Hamiltonian $(H_{3Din-hldn}^{slab-\left( 0,1,1 \right)})$ can be written as a 4$\times$T$\times$N-by-4$\times$T$\times$N matrix:

$$H_{3Din-hldn}^{slab-\left( 0,1,1 \right)}\left( k_{x} \right)=\left[ \begin{matrix} H_{0}{\left( k_{x} \right)|}_{l_{z}=1} & H_{couple} & \cdots& 0 & 0 \\ H_{couple}^{*} & H_{0}{\left( k_{x} \right)|}_{l_{z}=2} & \cdots& 0 & 0 \\ 0 & H_{couple}^{*} & \cdots& 0 & 0 \\ \vdots& \vdots& \ddots& \vdots& \vdots\\ 0 & 0 & \cdots& H_{0}{\left( k_{x} \right)|}_{l_{z}=N-1} & H_{couple} \\ 0 & 0 & \cdots& H_{couple}^{*} & H_{0}{\left( k_{x} \right)|}_{l_{z}=N} \end{matrix} \right] (S4)$$

Where $H_{0}$ and $H_{couple}$ are both 4$\times$T-by-4$\times$T matrices:

$$H_{0}(k_{x})=\left[ \begin{matrix} A\left( k_{x} \right) & B\left( k_{x} \right) & \cdots& 0 & 0 \\ B^{*}\left( k_{x} \right) & A\left( k_{x} \right) & \cdots& 0 & 0 \\ 0 & B^{*}\left( k_{x} \right) & \cdots& 0 & 0 \\ \vdots& \vdots& \ddots& \vdots& \vdots\\ 0 & 0 & \cdots& A\left( k_{x} \right) & B\left( k_{x} \right) \\ 0 & 0 & \cdots& B^{*}\left( k_{x} \right) & A\left( k_{x} \right) \end{matrix} \right] (S5a)$$

$$H_{couple}=\left[ \begin{matrix} t_{a} & 0 & \cdots& 0 & 0 \\ 0 & t_{b} & \cdots& 0 & 0 \\ \vdots& \vdots& \ddots& \vdots& \vdots\\ 0 & 0 & \cdots& t_{a} & 0 \\ 0 & 0 & \cdots& 0 & t_{b} \end{matrix} \right] (S5b)$$

In which:

$$A_{11}\left( k_{x} \right)=A_{33}\left( k_{x} \right)=+M+t_{2}e^{i\phi}e^{-ik_{x}}+t_{2}e^{-i\phi}e^{ik_{x}} (S6a)$$

$$A_{22}\left( k_{x} \right)=A_{44}\left( k_{x} \right)=-M+t_{2}e^{i\phi}e^{ik_{x}}+t_{2}e^{-i\phi}e^{-ik_{x}} (S6b)$$

Except for ${H_{0}}_{11}\left( k_{x} \right)=+M$ and ${H_{0}}_{4T 4T}\left( k_{x} \right)=-M$.

$$A_{12}\left( k_{x} \right)=A_{21}^{*}\left( k_{x} \right)=t_{1x}\left( l_{z} \right)(1+e^{ik_{x}}) (S6c)$$

$$A_{13}\left( k_{x} \right)=A_{31}^{*}\left( k_{x} \right)=t_{2}e^{-i\phi}+t_{2}e^{i\phi}e^{ik_{x}} (S6d)$$

$$A_{14}\left( k_{x} \right)=A_{41}^{*}\left( k_{x} \right)=0 (S6e)$$

$$A_{23}\left( k_{x} \right)=A_{32}^{*}\left( k_{x} \right)=t_{1y} (S6f)$$

$$A_{24}\left( k_{x} \right)=A_{42}^{*}\left( k_{x} \right)=t_{2}e^{-i\phi}+t_{2}e^{i\phi}e^{-ik_{x}} (S6g)$$

$$A_{34}\left( k_{x} \right)=A_{43}^{*}\left( k_{x} \right)=t_{1x}\left( l_{z} \right){(1+e}^{-ik_{x}}) (S6h)$$

$$B\left( k_{x} \right)=\left[ \begin{matrix} 0 & 0 & 0 & 0 \\ 0 & 0 & 0 & 0 \\ {t_{2}e^{i\phi}+t}_{2}e^{-i\phi}e^{-ik_{x}} & 0 & 0 & 0 \\ t_{1y} & {t_{2}e^{i\phi}+t}_{2}e^{-i\phi}e^{ik_{x}} & 0 & 0 \end{matrix} \right] (S6i)$$

The projected dispersions and the corresponding eigenfield distributions are shown in Figs. 2e, 2f, and 2g in the main text, which demonstrate the existence of chiral zeroth Landau levels and the one-sided chiral hinge states of Fermi arcs.

**(4) Refined inhomogeneous 3D Haldane model with layer-dependent interlayer coupling**

To establish a more accurate mapping between the TB model and the gyromagnetic photonic crystal, we construct a refined inhomogeneous 3D Haldane model (Fig. S4a) to incorporate layer-dependent interlayer coupling terms $t_{a}\left( l_{z} \right)$ and $t_{b}\left( l_{z} \right)$ into the supercell Hamiltonian as:

$$\left| t_{a}\left( l_{z} \right) \right|=\left| t_{b}\left( l_{z} \right) \right|=1.1\times sqrt[\frac{t_{1x}\left( l_{z} \right)}{t_{1x}\left( 1 \right)}] (S6)$$

where $l_{z}$*​* denotes the layer index along the *z* direction, and we preserve the core relation $t_{a}\left( lz \right)=-t_{b}(lz)$ with equal magnitudes for each layer, and set the interlayer coupling strength to decrease gradually along the z direction, matching the variation trend of the in-plane coupling ratio $t_{1x}/t_{1y}$. This layer-dependent modulation of interlayer couplings accounts for the rectangular deformation of the YIG rods in the photonic crystal, which alters the interlayer coupling strength across layers.

The updated TB results are presented in Figs. S4b and S4c. These figures show that the lower portions of the Landau level plateaus exhibit a slight tilt toward the lower-left direction, in excellent agreement with the tilted Landau levels observed in our full-wave electromagnetic simulations and experimental measurements of the 3D inhomogeneous gyromagnetic photonic crystal. Additionally, we observe a slight delocalization of the chiral hinge states within the eigenenergy distributions—denoted by the green and blue dots in Fig. S4c—along with partial field penetration into the bulk. This behavior also matches the characteristics of the measured one-sided chiral hinge states.


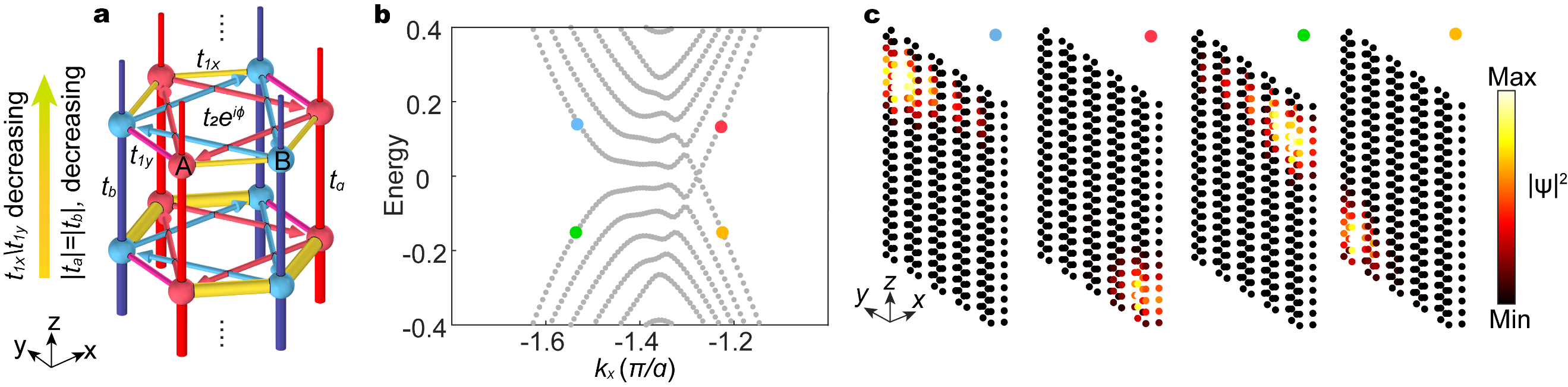


**Fig. S4 | Refined inhomogeneous 3D Haldane model with layer-dependent interlayer couplings. a** Schematic of the refined inhomogeneous 3D Haldane model with the strength of the interlayer couplings ( $\left| t_{a} \right|$and $\left| t_{b} \right|$, with $\left| t_{a} \right|=\left| t_{b} \right|$ and $t_{a}=-t_{b})$ decreasing along *z* direction, compared with the TB model shown in Fig. 2a in the main text. **b** The projected band dispersions along the $k_{x}$ direction for the refined TB model, in which the Landau levels are slightly tilted compared to Fig. 2f in the main text. **c** The calculated eigenenergy distributions of the one-sided chiral hinge states of Fermi arcs at two different energies corresponding to two pairs of colored dots in **b**.”

**Note 2.** **Modeling of gyromagnetic Weyl photonic crystals in COMSOL Multiphysics**

We now discuss the simulation details of the bulk band structures of the gyromagnetic Weyl photonic crystal in COMSOL Multiphysics. Figs. S5a and S5b present the frequency-dependent elements $\mu_{r}$ and $\kappa$ of the permeability tensor of the gyromagnetic material (YIG) for ${\mu_{0}H}_{0} =110mT$. The grey dashed lines indicate the Weyl frequency, which is far away from the gyromagnetic material’s resonant frequency; thus, the material dispersion within the operating frequency range is negligible.

In the numerical simulations, we choose a hexagonal unit cell of a gyromagnetic Weyl photonic crystal, consisting of a square YIG rod (gray) sandwiched between two permanent magnets (red and blue) standing on a perforated copper plate (yellow), as shown in Fig. S6a. To construct periodic boundary conditions along the z-direction, the copper plate is cut into two halves and moved so that one half is positioned above the top magnet, as shown in Fig. S6b. For simplicity, we replace the complementary region of copper plates and permanent magnets with air (gray color) and treat copper plates and permanent magnets as perfect electric conductor (PEC) boundary conditions, as illustrated in Fig. S6c. Finally, we apply PEC boundary conditions to the inner boundaries (Fig. S6d) and periodic boundary conditions to the outmost boundaries (Fig. S6e) to calculate the bulk band structure of the magnetic Weyl photonic crystals.


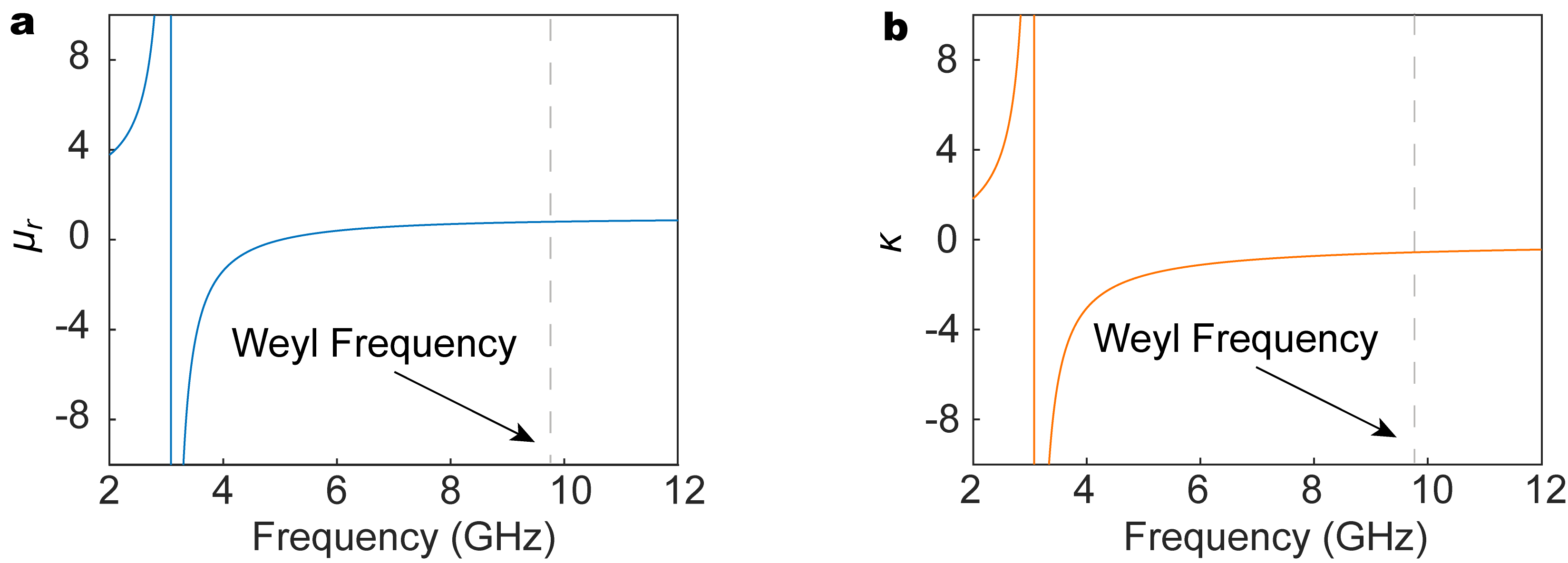


**Fig. S5 | Permeability tensor of the gyromagnetic material. a, b** Frequency-dependent elements $\mu_{r}$ and $\kappa$ of the permeability tensor of the gyromagnetic material for ${\mu_{0}H}_{0} =110mT$. The grey dashed lines in **a** and **b** indicate the Weyl frequency.


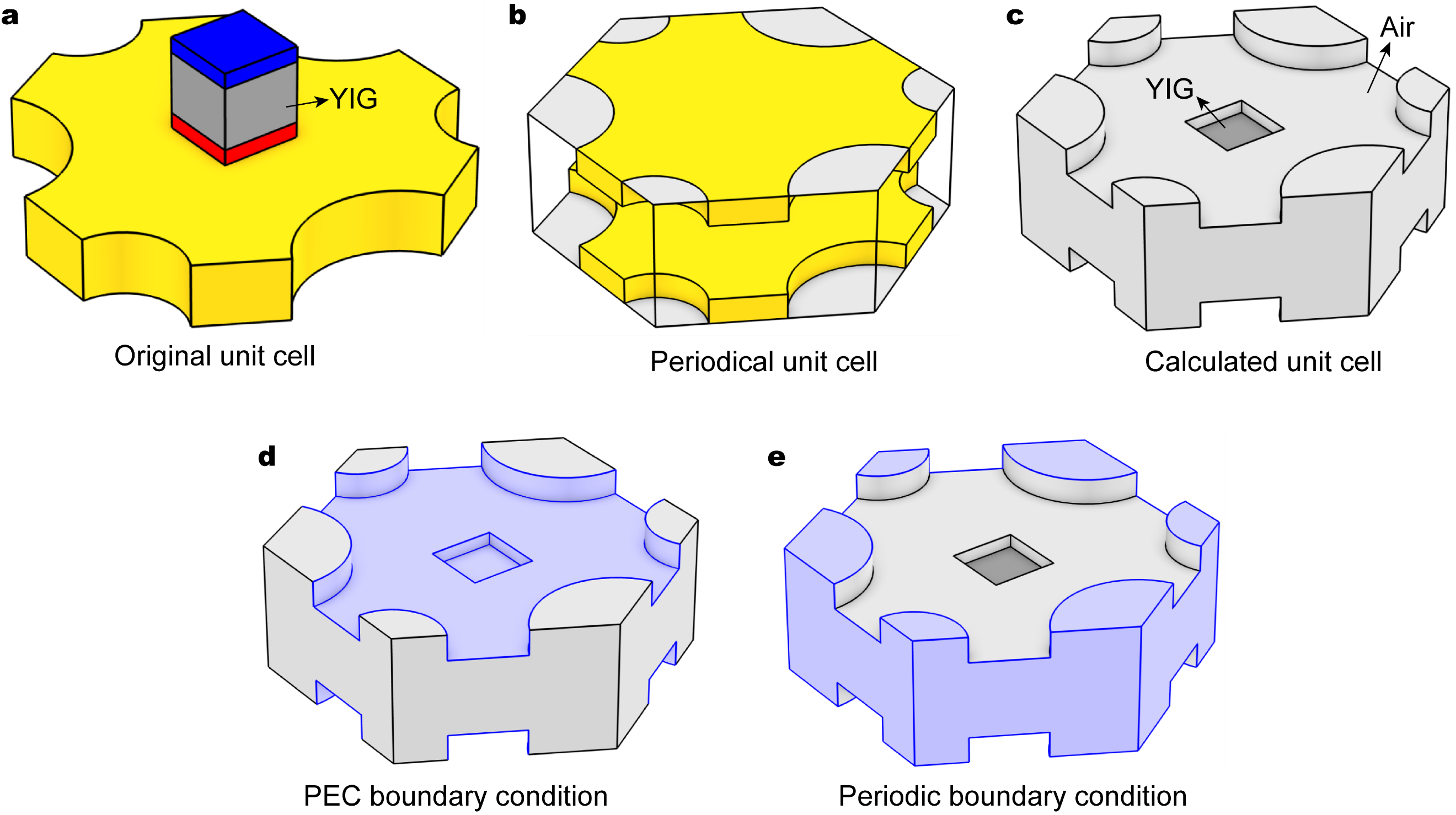


**Fig. S6 | Details of modeling of the magnetic Weyl photonic crystal in COMSOL Multiphysics. a** Original unit cell with the real structure. **b** The copper plate is sectioned into two halves, and one half is moved above the top magnet to construct a periodical unit cell. **c** The calculated unit cell comprising air and YIG materials. **d, e** Perfect electric conductor (**d**) and periodic (**e**) boundary conditions for the calculated unit cell.

**Note 3.** **Fermi arc surface states of the magnetic Weyl photonic crystal**

In this section, we study the Fermi arc surface states of the magnetic Weyl photonic crystal. We first construct a finite rectangular supercell with open boundary conditions in the *y* direction and periodic boundary conditions in the *x* and *z* directions, and calculate the projected Fermi arc surface state dispersions along the $k_{x}$ direction for $k_{z}= \pi/h$, $0.53\pi/h$, and 0, respectively, as shown in Figs. S7a-S7c. The blue dot represents the Weyl point at $(k_{x}, k_{y}, k_{z}) = (-1.332\pi/a, 0, 0.530\pi/h)$, the orange (purple) lines represent the Fermi arc surface state dispersions on the front (back) surfaces. Via a “wormhole” tunneling assisted by the Weyl points (blue and red dots), the two open surface Fermi arcs on opposite surfaces (orange and purple arcs) form a closed Fermi loop, which is essential to the 3D QHE of Fermi arcs, as shown in Fig. S7d. Figs. S7e-S7f show the field distributions of the Fermi arc surface states on the front (orange dot) and back (purple dot) surfaces, respectively, from which we can see that the Fermi arc surface states are tightly localized on the opposite surfaces and propagate in opposite directions.


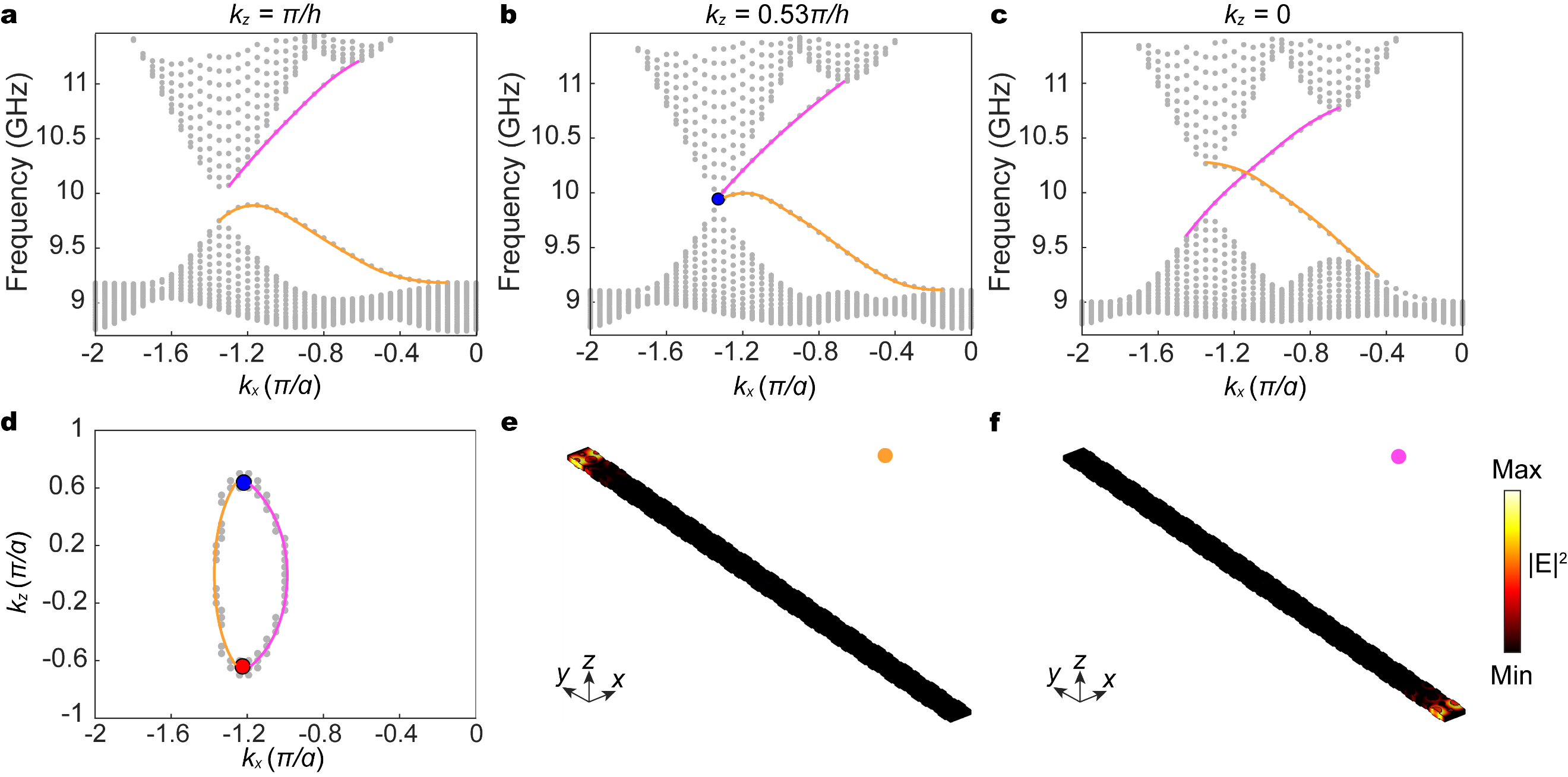


**Fig. S7 | Simulated band dispersion, closed Fermi loop, and eigen-field distributions of the** **Fermi arc surface states of the magnetic Weyl photonic crystal. a-c** Projected dispersions of the Fermi arc surface states (purple and orange lines) along the $k_{x}$ direction for a finite rectangular supercell with open boundary conditions in the *y* direction and periodic boundary conditions in the *x* and *z* directions for $k_{z}= \pi/h$ (**a**), $0.53\pi/h$ (**b**), and 0 (**c**), respectively. The blue sphere in **b** represents the Weyl point. The surface-state dispersions for the front and back surfaces are indicated by orange and purple lines, respectively. **d** The iso-frequency contour of the front (orange) and back (purple) Fermi arc surface states at the Weyl frequency. The two open Fermi arcs (orange and purple) on opposite surfaces formed a closed Fermi loop. **e, f** Eigen-field distributions of the Fermi arc surface states for the front (orange dot) and back (purple dot) surfaces with open boundary conditions in the *y* direction and periodic boundary conditions in the *x* and *z* directions.

**Note 4.** **Landau Level formation and boundary modification**

In this section, we provide an intuitive understanding of Landau level formation and demonstrate a crucial step toward realizing Landau plateaus and one-sided chiral hinge states of Fermi arcs in an inhomogeneous magnetic Weyl photonic crystal. As illustrated in Fig. S8a, the 3D inhomogeneous structure with $t_{1x}/t_{1y}$ decreasing along the *z* direction (Fig. 2a) can be effectively modeled as a series of vertically coupled two-dimensional (2D) layers. Each layer exhibits boundary band dispersion (colored lines) along the $k_{x}$ direction under periodic boundary conditions. In the presence of PMF, the boundary band dispersions shift leftward ($-k_{x}$ direction) among layers, as shown in Fig. S8b, with the same moving direction as the Weyl points under PMF. The interlayer couplings $t_{z}$ open band gaps near the band crossing regions (black dashed circles) and generate the Landau levels (black lines) shown in Fig. S8c^1^. If the band-crossing regions have slightly different energies, the Landau levels will be tilted.


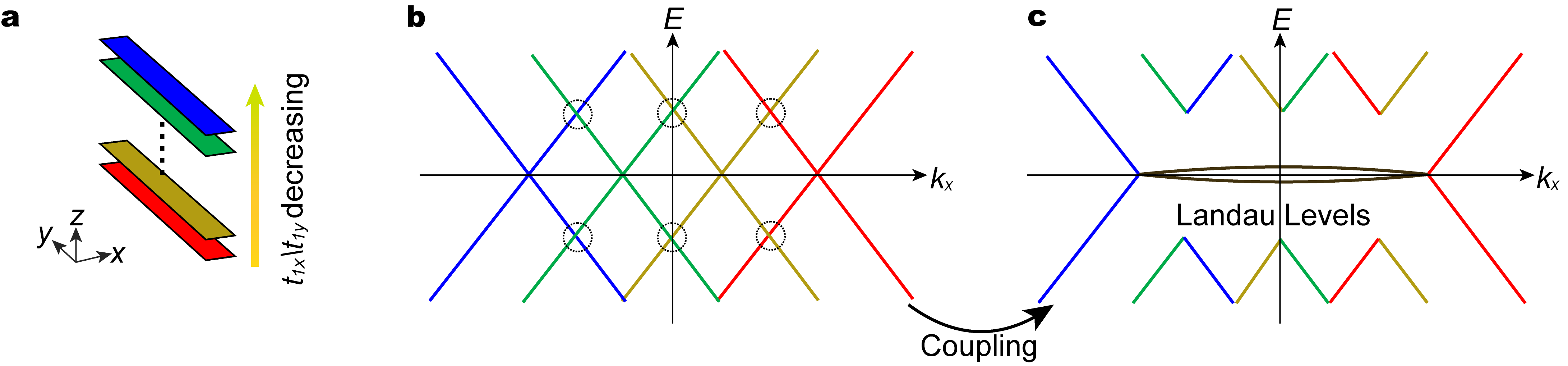


**Fig. S8 | An intuitive physical picture of the formation of Landau levels**. **a** A 3D inhomogeneous structure with gradient $t_{1x}/t_{1y}$ ratios among vertically coupled layers. Each layer is a finite rectangular supercell with open boundary conditions in the *y* and *z* directions and periodic boundary conditions in the *x* direction. **b** The boundary band dispersions (colored lines) along the $k_{x}$ direction for each layer, which shift leftward as a result of the PMF. **c** The interlayer couplings between the boundary modes along the *z* direction open gaps near the band cross regions (black dashed circles in **b)**, resulting in a band structure with the emergence of Landau levels (black lines).

This intuitive physical picture describing the formation of Landau levels is well supported by the TB model. Fig. S9a shows the projected TB dispersion of a simplified supercell (with fewer layers along the z direction) compared to that in Fig. 2f of the main text. Fig. S9b presents the eigenfield distributions of the chiral hinge states of Fermi arcs corresponding to the colored dots in Fig. S9a, which show that the eigenstates marked by the dots in the same color exist in identical layers and positions (e.g., the two eigenstates indicated by the two red dots are localized at the upper-right corner). We use colored lines to connect the same-colored dots, with distinct colors representing boundary band dispersions of different layers (e.g., eigenstates marked by red and blue (green and orange) dots both appear at the upper (lower) layers, and thus the blue (red) lines connecting them represent the boundary band dispersions of the upper (lower) layers). Consequently, the crossed blue lines appear at the left of the crossed red lines, consistent with the WP moving direction (from the bottom to the top layer, the WPs move along the $k_{x}$ direction). These crossed colored lines can be viewed as shifted boundary-band dispersions for each layer, which intersect with one another, and the interlayer couplings open gaps near the band crossings (black dashed circles) and give rise to the Landau levels.

Unfortunately, real magnetic photonic crystals exhibit significant deviations from this ideal scenario. Unlike the TB model, deforming the square gyromagnetic rods into rectangles in magnetic Weyl photonic crystals not only affects the $t_{1x}/t_{1y}$ ratios but also modifies the inter-layer couplings $t_{a}$ and $t_{b}$, which therefore shifts the Weyl frequencies and the frequency of the boundary band dispersions for each layer. Fig. S9c displays the projected dispersions of a bilayer rectangular supercell with *m* = 0.7 (top layer) and *m* = 1 (bottom layer) along the $k_{x}$ direction. The eigenfield distributions in Fig. S9d show that crossed blue lines (*m* = 0.7) appear above (rather than at the left with the same frequency at the crossing point) the crossed red line (*m* = 1), indicating that gyromagnetic rod deformation increases the Weyl frequency and consequently shifts the boundary band dispersions upward, which fails the interlayer couplings and band gap opening. For comparison, Figs. S9e-S9f show the dispersions of a bilayer rectangular supercell with *m* = 1.3 (top layer) and *m* = 0.7 (bottom layer), from which we can see that the red crossed lines lie above their blue counterparts, further verifying the Weyl frequency shifting in the deformed magnetic Weyl photonic crystal. Given the opposite Weyl frequency offsets in the *m < 1* and *m > 1* cases, we chose the deformed magnetic Weyl photonic crystals with *m* < 1 for our experimental realization, as mentioned in the main text.


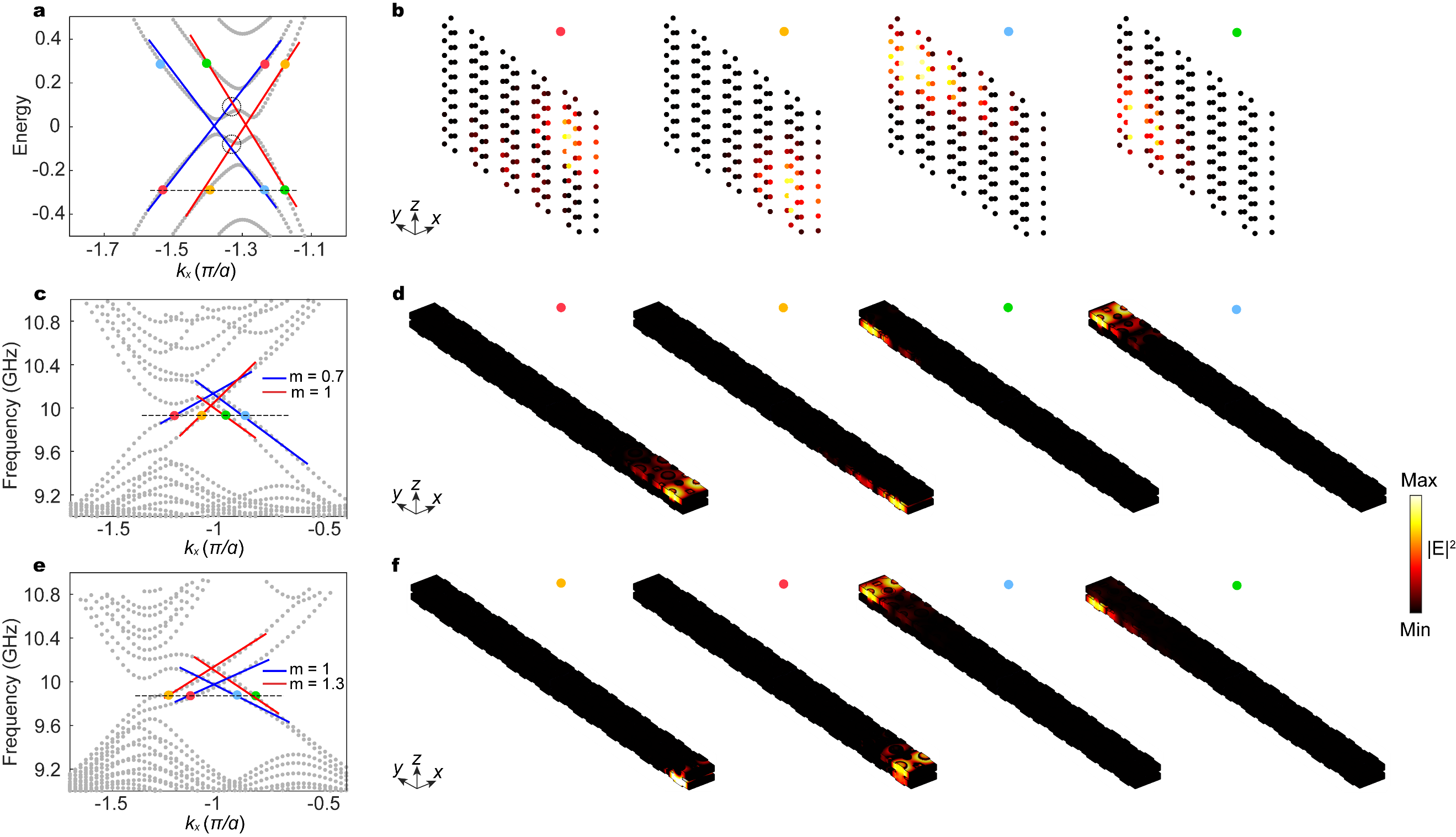


**Fig. S9 | a, c, e** Projected dispersions along the $k_{x}$ direction for the TB model (**a**), deformed magnetic Weyl photonic crystals with *m* < 1 (**b**), and *m* > 1 (**c**). The colored crossed lines represent boundary band dispersions of deformed magnetic Weyl photonic crystals in different layers (blue line: lower layer; red line: upper layer). **b, d, f** Electric field distributions of the eigenstates for the TB model (**b**), deformed magnetic Weyl photonic crystals with *m* < 1 (**d**), and *m* > 1 (**f**), corresponding to the colored dots in **a, c,** and **e.**

To make the blue crossed lines lie at the left of the red crossed lines with almost the same frequencies for the boundary band dispersions in the deformed magnetic Weyl photonic crystals, we adopt boundary modification to tune the boundary band dispersion of each layer of the deformed magnetic Weyl photonic crystals. Although boundary modification cannot shift WPs in frequency, as WPs are bulk properties, it can effectively tune the boundary band dispersions. Fig. S10 illustrates the boundary modification scheme used to align the magnetic Weyl photonic crystal simulation results with the TB model results by adjusting the radii of the outermost air holes on the front surface. As shown in Fig. S10a, the radii of the small and large air holes are $r_{x}$ and $r_{t}$, respectively, with $r_{t}=n*r_{x}$. Consequently, the dispersion of the Fermi arc surface state at the front surface can be tuned by varying *n*. The projected dispersions of the Fermi arc surface states along the $k_{x}$ direction with different *n* (red, orange, and blue dots) are shown in Fig. S10b, from which we can see that the boundary dispersion of the front surface moves down as we increase the value of *n*. The electric field distributions of the Fermi arc surface states are shown in Fig. S10c, from which we can see that the surface arc states are tightly localized on the surface.


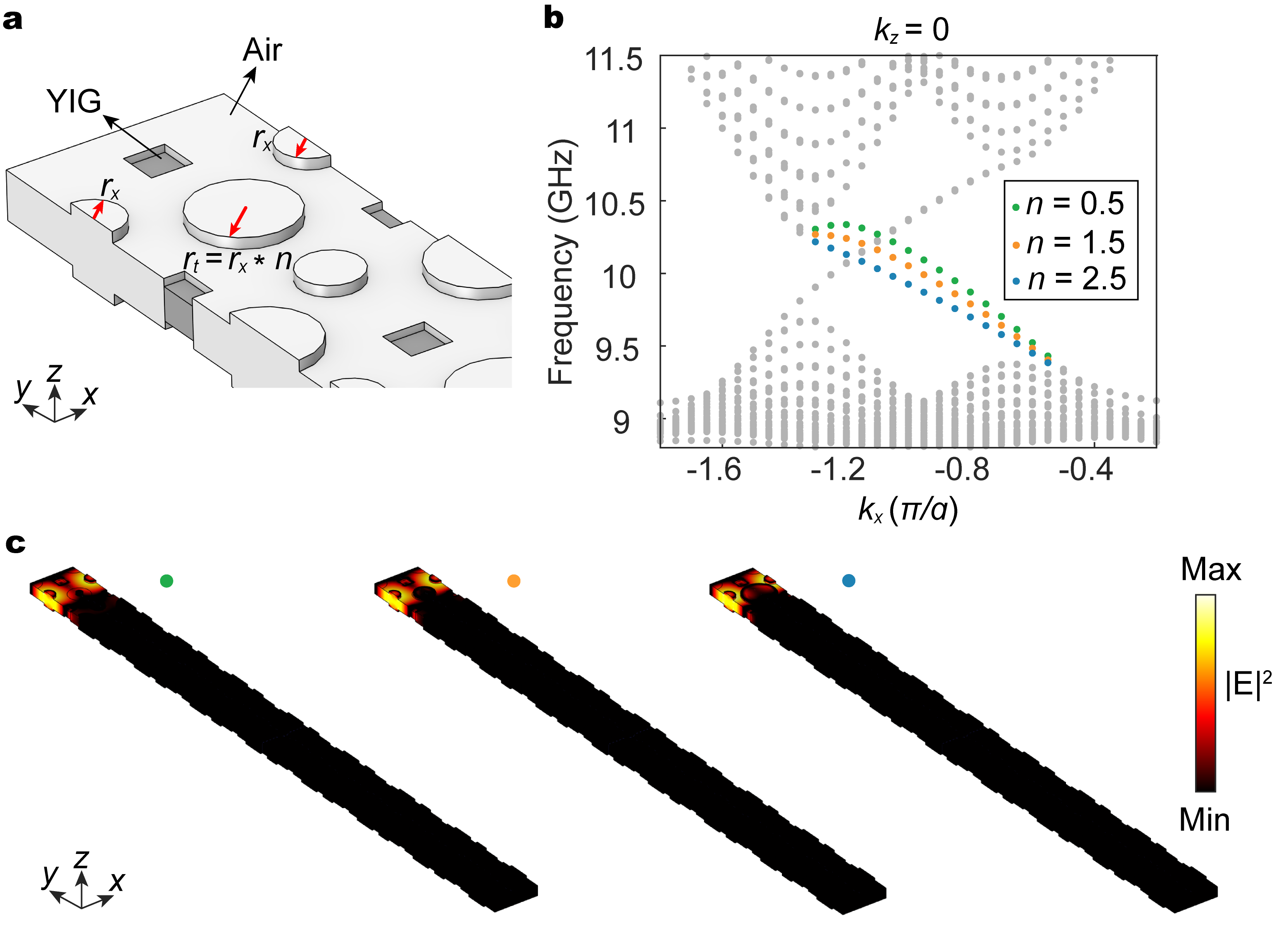


**Fig. S10 | a,** Illustration of the outermost air holes on the front surface, with $r_{x}$ and $r_{t}$ as the radii of the small and large air holes. **b,** Projected dispersion along $k_{x}$ with different parameter *n.* **c,** Eigen field distributions of the surface states on the front surface, corresponding to different parameter *n* in **b**.

**Note 5. Simulated eigen-field distributions of the one-sided chiral hinge states of Fermi arcs**

Fig. S11 shows the simulated band dispersions and electric field distribution of the one-sided chiral hinge states of Fermi arcs without (Figs. S11a-S11c) and with (Figs. S11d-S11f) boundary modification. As shown in Fig. S11a, without boundary modification, Landau levels fail to form because the uncoupled boundary-band dispersions simply intersect. Additionally, the chiral hinge states emerge on the top edges of both the front and back surfaces (Figs. S11b-S11c), rather than the desired one-sided chiral hinge states of Fermi arcs localized on the diagonal edges of the front and back surfaces. In contrast, with boundary modification, a series of Landau plateaus (purple dashed lines) emerges, as shown in Fig. S11d, and the one-sided chiral hinge states of Fermi arcs can be clearly seen in their simulated electric field distributions, which are mainly localized on the diagonal edges of the front and back surfaces (Figs. S11e-S11f). These results show that the boundary modification is critical for the formation of the chiral Landau level plateaus and the one-sided chiral hinge states of Fermi arcs.


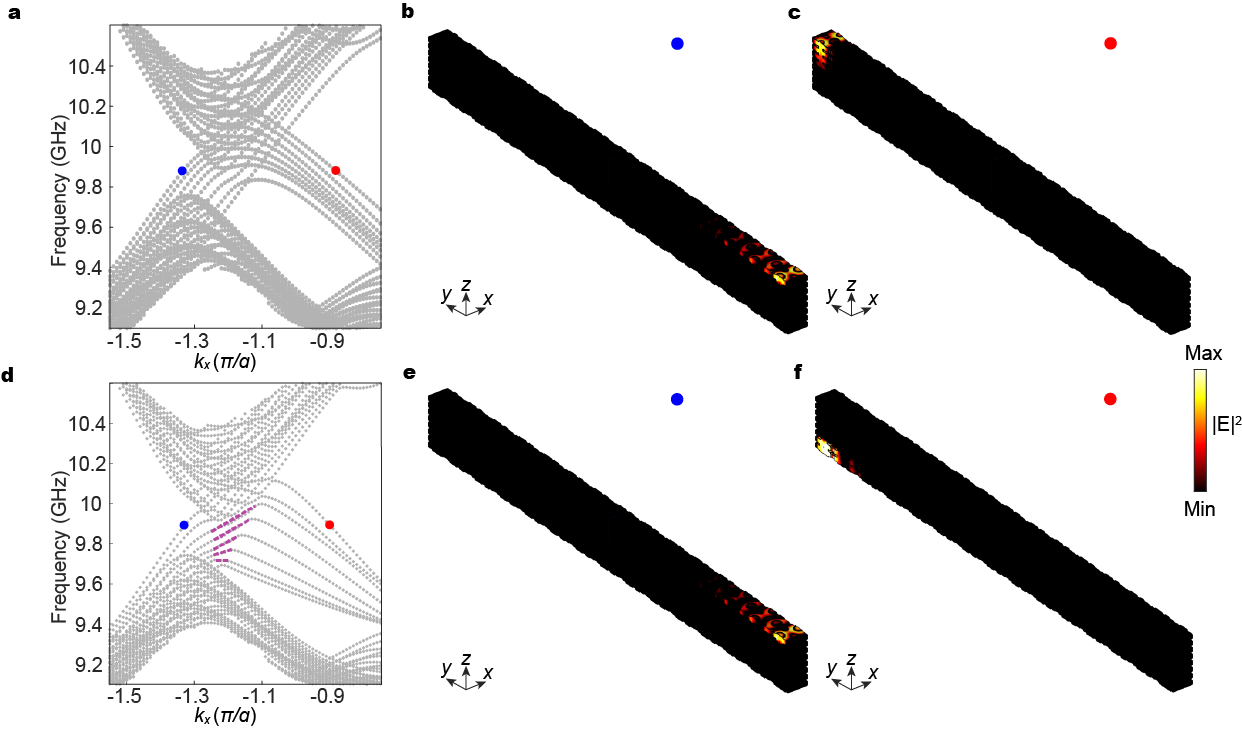


**Fig. S11 | Simulated band dispersions and eigen-field distributions of chiral hinge states without and with boundary modification. a, d** Projected band dispersions along the $k_{x}$ direction for a finite rectangular supercell structure without (**a**) and with (**d**) boundary modifications. The supercell has open boundary conditions in the *y* and *z* directions and periodic boundary conditions in the *x* direction. **b**, **c** Simulated eigen-field distributions of the chiral hinge states without boundary modification, both of which are localized on the top edges of the front and back surfaces. **e**, **f** Simulated eigen-field distribution of the chiral hinge states with boundary modification, which are localized at the diagonal edges of the front and back surfaces.

**Note 6. Propagation behavior of the one-sided chiral hinge states on the *y-z* surface**

To examine the propagation behavior of the one-sided chiral hinge states on the *y-z* surface, we replace the absorbing boundaries with open boundaries in both the TB and the real gyromagnetic photonic crystal. As shown in Figs. S12a-b, the chiral hinge states do not vanish or backscatter upon reaching the *y-z* surface; instead, they continue to propagate along the hinges.

To understand this behavior, we compute the $k_{y}$​-projected band structure of a finite structure with periodic (open) boundary conditions in the *y* (*x* and *z*) direction, as shown in Fig. S12c. The results reveal that Fermi arc surface states (Fig. S12d) exist on the *y-z* surface, similar to those in 3D Weyl photonic crystals in Ref. [53] in the main text. Unlike Ref. [53], our system lacks periodicity in the *z* direction due to the structural gradient, resulting in weak dispersion in that direction. Thus, when the one-sided chiral hinge states encounter the y-z surface, they couple to these Fermi arc surface states localized at the hinges and continue to propagate.


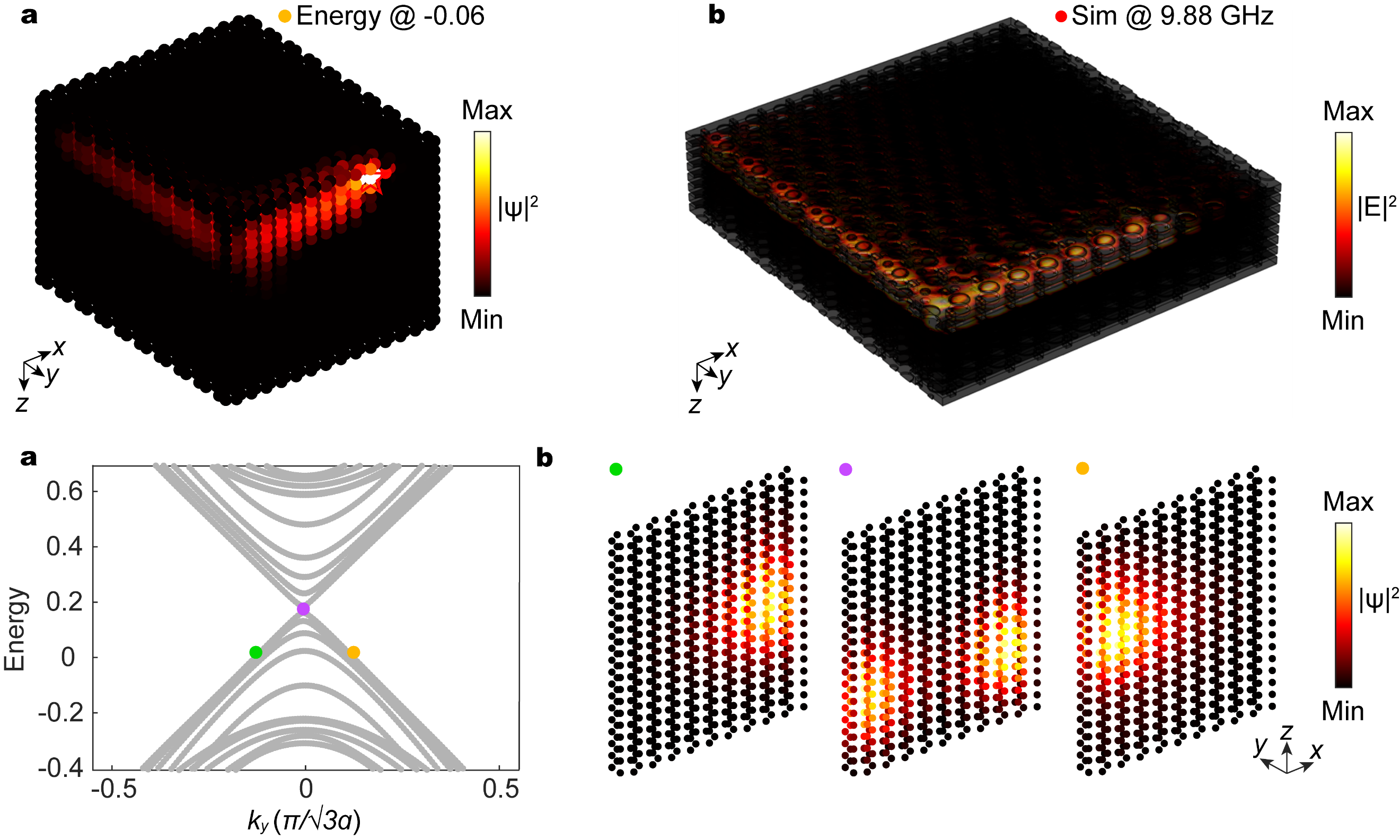


**Fig. S12 | a** Calculated energy distribution of the one-sided chiral hinge states in the TB model with open boundaries on the *y−z* surface at energy$=-0.06$. **b** Full-wave simulated electric field distribution of the one-sided chiral hinge states with open boundaries on the *y−z* surface at 9.88 GHz. **c** The projected band dispersions along the $k_{y}$ direction for a rectangular structure with periodic boundary conditions along the y direction and finite size in the *x−z* plane. **d** The calculated eigenenergy distributions of the Fermi arc surface states corresponding to the colored dots in **c**.

Notably, because the PMF is parallel to the $y$-$z$ planes and exerts no influence on the Fermi arc surface states, thus the one-sided chiral hinge state of Fermi arcs does not exist on the $y$-$z$ surfaces. All observed modes on the $y$-$z$ surface are Fermi arc surface states, as confirmed by the eigenmode analysis in Fig. S12. Owing to the gradient of the structure in the $z$-direction, these Fermi arc surface states are strongly localized and propagate collimated in specific $z$ regions, as shown in Fig. S13.


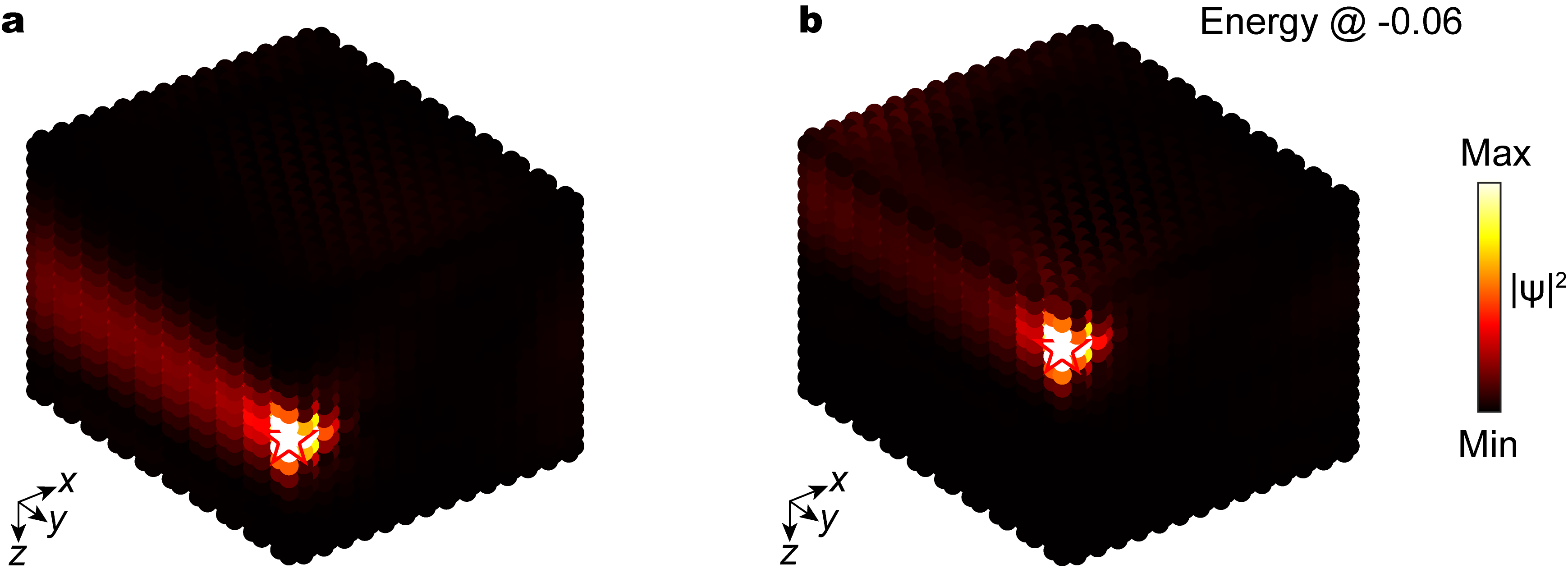


**Fig. S13 |** **Excitation of Fermi-arc surface states on the *y−z* surface at two distinct positions.**

**Note 7 Switching the one-sided chiral hinge states of Fermi arcs by reversing the PMF**

Finally, we show that the one-sided chiral hinge states of Fermi arcs can be switched to the other pair of diagonal hinges at the front and back surfaces by reversing the direction of PMF. This switching behavior originates from the inverted structural gradient upon PMF reversal, which reverses the momentum-space shifting direction of the Weyl points (from -$k_{x}$ to +$k_{x}$) and the corresponding boundary-state dispersions of each vertically stacked layer (as detailed in Supplementary Information Note 4). This inversion of the boundary-state dispersion shift relocates the chiral hinge states between opposite boundaries within the same x−z plane, thereby switching the one-sided chiral hinge states between the two pairs of diagonal hinges. Fig. S14a shows the projected band dispersions of a rectangular structure (which is finite in the *y*-*z* plane and periodic along the *x* direction) with $t_{1x}/t_{1y}$ increases along the *z* direction, which reverses the PMF, in contrast with the case discussed in Fig. 2 in the main text. For each specific energy, the one-sided chiral hinge states of Fermi arcs switched to the opposite sides of the front and back surfaces, as shown in Fig. S14b.

Fig. S14c shows the simulated projected band dispersions of the deformed magnetic Weyl photonic crystals with a reversed PMF along the $k_{x}$ direction, from which we can see the presence of Landau levels (purple dashed lines). Figs. S14d-S14e show the simulated eigen-field distributions of the one-sided chiral hinge states of Fermi arcs corresponding to the blue and red dots in Fig. S14c, which match well with the results for the green and yellow dots in Fig. S14b.

Fig. S14f shows a schematic of the system with a reversed PMF (green arrow) induced by increasing the parameter *m* along the *z* direction in the deformed magnetic Weyl photonic crystal. The one-sided chiral hinge states of Fermi arcs (red and purple arrows) are mainly localized at the top (bottom) of the front (back) surface as a result of the reversed PMF and propagate along the same direction as the case in Fig. 3e in the main text, because the chirality of the Fermi arc surface states remains unchanged. The simulated electric field distributions of the one-sided chiral hinge states of Fermi arcs at 9.88 GHz are shown in Figs. S14g-S14h, which are consistent with the results in the TB model and eigenstate simulation.


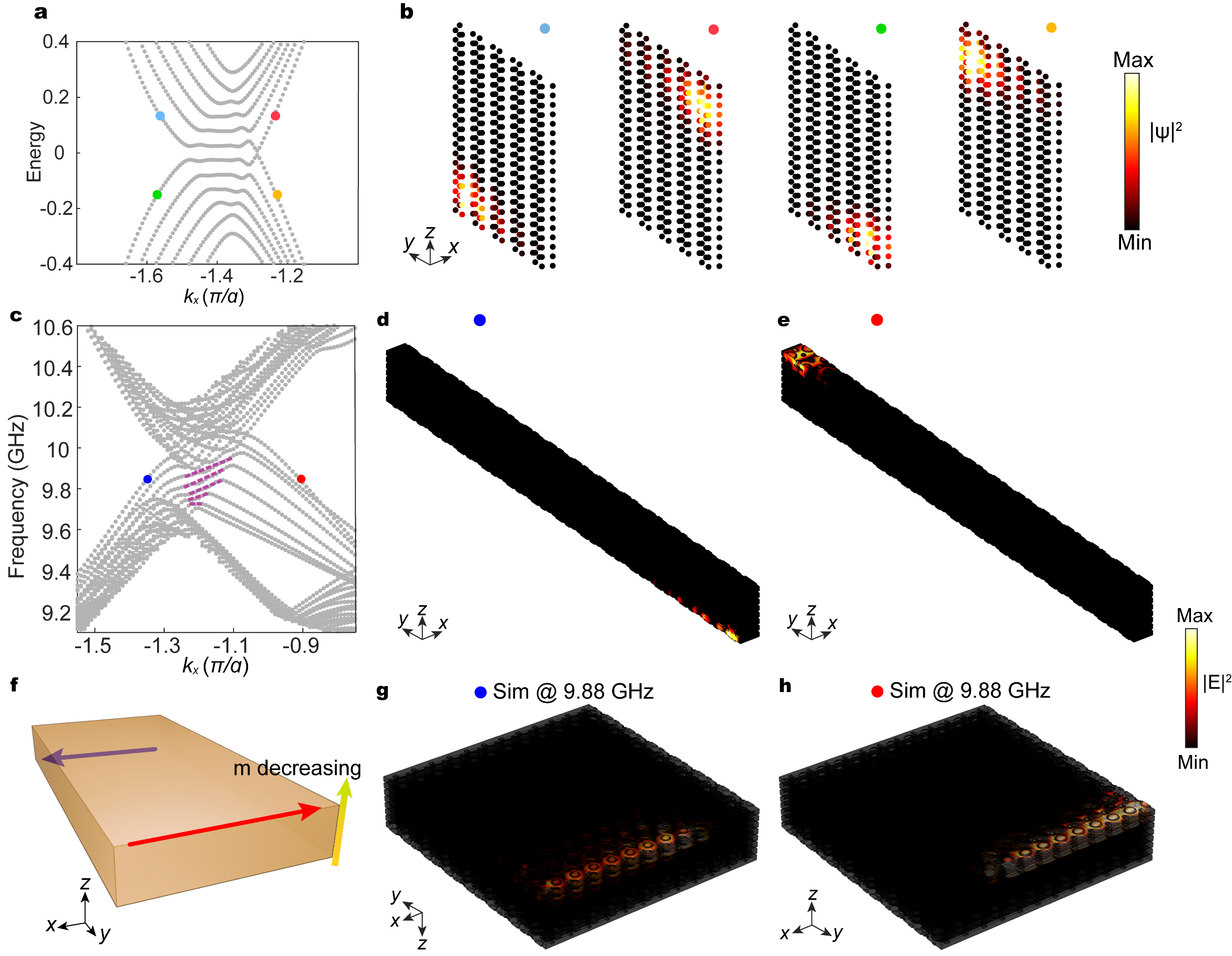


**Fig. S14| Switching the** **one-sided chiral hinge states of Fermi arcs by reversing the PMF. a** The projected band dispersions of the Landau levels along the $k_{x}$ direction with $t_{1x}/t_{1y}$ increases along the *z* direction. **b** The calculated eigenenergy distributions of the one-sided chiral hinge states of Fermi arcs at two different energies corresponding to two pairs of colored dots in **b**. **c** Projected band dispersions along the $k_{x}$ direction for a finite rectangular supercell structure (open boundary conditions in the *y* and *z* directions and periodic boundary conditions in the *x* direction) with *m* decreasing along the *z* direction. The purple dashed lines represent the Landau levels, and the blue and red dots represent the one-sided chiral hinge states of Fermi arcs. **d**, **e** Simulated eigen-field distribution of the chiral hinge states corresponding to the colored dots in **c**. The one-sided chiral hinge states are switched to the opposite sides of the front and back surfaces. **f,** Schematic of the structure with reversed PMF (green arrow) generated by *m* decreasing along the *z* direction (yellow arrow). The red and blue arrows represent the one-sided chiral hinge states of Fermi arcs localized at the other pair of diagonal hinges. **g**, **h** Simulated electric field distribution of the chiral hinge states of Fermi arcs corresponding to the colored dots in **c** at 9.88 GHz.

**References**

1. Zhang, Z. Q. *et al.* Three-dimensional quantum anomalous Hall effect in Weyl semimetals, arXiv.2501.01399 (2025).
